# Dermatan Sulfate Affects the Activation of the Necroptotic Effector MLKL in Breast Cancer Cell Lines via the NFκB Pathway and Rac-Mediated Oxidative Stress

**DOI:** 10.3390/biom14070829

**Published:** 2024-07-10

**Authors:** Grzegorz Wisowski, Adam Pudełko, Monika Paul-Samojedny, Katarzyna Komosińska-Vassev, Ewa M. Koźma

**Affiliations:** 1 Department of Clinical Chemistry and Laboratory Diagnostics, Faculty of Pharmaceutical Sciences in Sosnowiec, Medical University of Silesia, Jedności 8, 41-200 Sosnowiec, Poland; a.pudelko@gyncentrum.pl (A.P.); kvassev@sum.edu.pl (K.K.-V.); mkozma@sum.edu.pl (E.M.K.); 2 Department of Medical Genetics, Faculty of Pharmaceutical Sciences in Sosnowiec, Medical University of Silesia, Jedności 8, 41-200 Sosnowiec, Poland; mpaul@sum.edu.pl

**Keywords:** dermatan sulfate, breast cancer, MLKL, NFκB transcription factor, Rac GTPase, oxidative stress, cell death

## Abstract

Dermatan sulfate (DS) is a glycosaminoglycan characterized by having a variable structure and wide distribution in animal tissues. We previously demonstrated that some structural variants of DS were able to rapidly induce moderate necroptosis in luminal breast cancer cells when used at a high concentration. We have now investigated the mechanisms underlying the DS-mediated activation of the necroptotic executor MLKL using immunofluorescence, Western blotting and pharmacological inhibition. The two main processes, by which DS influences the phosphorylation of MLKL, are the activation of NFκB, which demonstrates a suppressive impact, and the induction of oxidative stress, which has a stimulatory effect. Moreover, the triggering of the redox imbalance by DS occurs via the modulatory influence of this glycosaminoglycan on the rearrangement of the actin cytoskeleton, requiring alterations in the activity of small Rho GTP-ase Rac1. All of these processes that were elicited by DS in luminal breast cancer cells showed a dependence on the structure of this glycan and the type of cancer cells. Furthermore, our results suggest that a major mechanism that is involved in the stimulation of necroptosis in luminal breast cancer cells by high doses of DS is mediated via the effect of this glycan on the activity of adhesion molecules.

## 1. Introduction

Dermatan sulfate (DS) is an unbranched copolymeric glycosaminoglycan (GAG) that is composed of two types of disaccharide units. Each of these units contains N-acetylgalactosamine (GalNAc) residue but differs in regard to hexuronate residue, which can be glucuronate (GlcA) or its 5C epimer iduronate (IdoA) [1]. The majority of GalNAc residues as well as some hexuronate ones are modified by sulfation, which occurs on the hydroxyl group at the 4 and/or 6C or 2C position, respectively. These modifications, which are characterized by a variable pattern, promote a great reactivity of DS, facilitating its ionic interactions with many protein binding partners including growth factors, cytokines, cell surface receptors, adhesion molecules, enzymes or their effectors as well as the so-called structural proteins/glycoproteins of the extracellular matrix (ECM) [1]. However, the binding profile of DS is variable depending on the variable sulfation and GlcA to IdoA epimerization patterns of this GAG, which causes the modulation of the biological functions of its binding partners. The above-mentioned characteristics of DS could position this GAG as a significant regulator of different biological processes. This possibility is further supported by the wide distribution of the GAG in animal tissues as a component of the ECM and cell-surface-located glycoproteins, which are called proteoglycans (PGs) [2].

Tumorigenesis is a process that is associated with significant alterations in the DS (and DSPGs) metabolism [3]. However, both the importance of the structural remodeling of DS and the consequences of increased DSPG degradation in the tumor niche that leads to an increased influx of DS to the cancer cell surface are poorly known. Our previous study showed [4] that the structural variants of DS that originate from both non-neoplastic human tissues (normal fascia lata and fibrosis-affected palmar fascia) and porcine intestinal mucosa were able to rapidly induce moderate necroptosis in cultured luminal breast cancer cells when used at a high concentration, although the mechanisms that are responsible for this effect are unknown. Interestingly, the above-mentioned human variants of DS share some structural features with the GAG from the tumor microenvironment, such as a high content of unsulfated disaccharides and disaccharides with 6-O-sulfated GalNAc residue as well as a high proportion of glucuronate-containing disaccharides in the glycan chain composition [3,4].

Necroptosis is a lytic form of regulated cell death, which releases lysosomal components via the activation of lysosomal exocytosis, thereby generating a damage-associated molecular pattern and triggering the inflammatory process [5]. Despite the fact that necroptosis is not necessary for normal embryogenesis, development and homeostasis, this death is an important mechanism in various processes, including pathological events, such as viral infections; chronic inflammation and fibrosis; acute injuries of the heart, kidney, lung and liver; neurodegenerative diseases, metabolic diseases as well as chemotherapy [6]. Several extra- or intracellular stimuli can induce necroptosis including (1) the activation of various receptors such as death receptors, e.g., tumor necrosis factor (TNF) receptor 1, as well as some Toll-like receptors (TLRs) or interferon receptors [6]; (2) the ligation of some adhesion molecules, e.g., CD11b and CD18 belonging to the integrin family [7] or disturbances in the integrin-linked kinase-mediated transduction of signals from the integrin receptors [8]; (3) metabolic events such as the mitochondrial overproduction of reactive oxygen species (ROS) [9] or the activation of the Hippo/YAP pathway [10]. However, the intracellular signaling or events that lead to the necroptosis induction are not yet fully clarified. Interestingly, the intracellular pathways, which are triggered via the activation of the TNFR1 death receptor or TLR4, serve to induce pro-survival signals by stimulating the NFkB activation, and the start of necroptosis is a rare effect of their initiation [5]. Nevertheless, independent of the driving mechanisms, two elements are crucial for necroptosis to occur. The first is the receptor-interacting protein kinase 3 (RIPK3), belonging to the RIP homotypic interaction motif-containing proteins, which upon activation participates in the formation of a necrosome [5]. This latter structure provides a platform for the recruitment of the mixed lineage kinase domain-like pseudokinase (MLKL) and its activation by phosphorylation at threonine 357 and serine 358 [5]. When activated, MLKL undergoes oligomerization and translocation to the plasma membrane, where it executes necroptosis via a membrane rupture [5].

DS is able to affect several of the above-mentioned factors, which can regulate the induction of necroptosis. In contrast to chondroitin sulfate (CS), which is structurally related to DS, this latter GAG has been shown to stimulate the activation of the NFκB pathway in some cells [11]. Moreover, DS can trigger oxidative stress in exposed luminal breast cancer cells [4]. This redox imbalance is also observed in vitro and in vivo in cells, which accumulate DS due to the genetically conditioned defects in the lysosomal degradation of this GAG [12,13]. Furthermore, two DS-containing proteoglycans, decorin and biglycan, can cause the rearrangement of the actin cytoskeleton in fibroblasts as well as transiently stimulating the activity of RhoA and Rac1 in them [14], which are small GTP-ases that are major regulators of cytoskeleton changes [15]. Thus, these results suggest that both PGs could affect the activity of integrins, which directly control the actin cytoskeleton architecture. All of these reports prompted us to explore whether the DS variants of a known structure that had previously been shown to quickly induce necroptosis and/or significantly reduce the viability of luminal breast cancer cells could influence the activity of NFκB, cause a rapid redox imbalance as well change the actin cytoskeleton arrangement in those cells. Furthermore, we wanted to elucidate whether such DS-induced events could underlie the DS-dependent activation of the necroptotic effector MLKL. In addition, we also aimed to investigate whether the tested DS variants could affect the RIPK3 gene expression.

## 2. Materials and Methods

### 2.1. The Structural Variants of DS

DS variants from the porcine intestinal mucosa (PM; Cat# C4384, Sigma-Aldrich, St. Louis, MO, USA), human fibrosis-affected palmar fascia (DF) and normal human fascia lata (NF) were used in the present study. The study protocol was approved by the local Bioethics Committee of the Medical University of Silesia in Katowice (permission no. PCN/CBN/0052/KB1/52/22). The procedures for isolating and purifying the human variants as well as their structural analysis were described previously [4].

### 2.2. Cell Lines

The luminal breast cancer cell lines BT-474 (HTB-20) and T47D (HTB-133) were obtained from the American Type Culture Collection. The cells were cultured, respectively, in DMEM/F12 (Cat# D8437, Sigma-Aldrich, St. Louis, MO, USA) or an RPMI-1640 (Cat# R8758, Sigma-Aldrich, St. Louis, MO, USA) medium that had been supplemented with 10% fetal bovine serum (FBS; Cat# S181H, Biowest, Riverside, MO, USA), MycoZap Plus-CL (Cat# VZA-2012, Lonza, Rockville, MD, USA) and insulin (5 μg/mL for BT-474 and 10 μg/mL for T47D) (Cat# BE02-033E20, Lonza, Rockville, MD, USA). The cells were grown at 37 °C in a 95% humidified atmosphere with 5% CO_2_ until they reached at least 80% confluency. They were then used.

### 2.3. Analysis of the DS-Mediated Activation of MLKL by Immunofluorescence

The BT-474 and T47D cells were seeded at a density of 6500 cells per well into eight-well glass chamber slides (Cat# 154534, Thermo Fischer Scientific, Rockville, MD, USA). After 24 h of incubation in the complete medium, the cells were transferred to a medium that had been supplemented with 0.5% FBS and allowed to grow for another 24 h. The cells were then exposed to a growth medium containing the tested DS variants at a concentration of 25 µg/mL for specified time periods as indicated in Section 3. Subsequently, the cells were washed three times with PBS, fixed with 3.7% formaldehyde for ten min, permeabilized in 0.3% Triton X-100 for 15 min and blocked in PBS containing 3% bovine serum albumin (BSA) and 0.3% Triton X-100 (PBS-Trit buffer) for one hour at 21 °C. Then, the cells were incubated with the rabbit monoclonal anti-phospho-MLKL (S358) antibody (Cat# ab187091, Abcam, Cambridge, UK) in PBS-Trit overnight at 4 °C. The next step was an incubation with Alexa Fluor Plus 555-conjugated goat polyclonal anti-rabbit IgG (Cat# A32732, Thermo Fisher Scientific, Rockville, MD, USA) for one hour at room temperature. Finally, the cells were stained for 7 min with 1 µg/mL solution of Hoechst 33342 (Cat# H3570, Thermo Fisher Scientific, Rockville, MD, USA) and captured using a Leica DMI 6000B microscope (Leica Microsystems GmbH, Wetzlar, Germany). The images were quantified using Leica AS hardware version 3.2.1.9702 (Wetzlar, Germany). In some of the experiments, before the DS was applied, the cells were preincubated for three hours with 20 µM cardamonin (Cat# 2509, Tocris, Bristol, UK), 6 or 12 µM EHop 016 (Cat# 6248, Tocris, Bristol, UK), 30 µM rhosin hydrochloride (Cat# 5003, Tocris, Bristol, UK) or 1 mM N-acetylcysteine amide (NACA; Cat# 5619, Tocris, Bristol, UK). Then, the cells were treated for 3.5 h with a combination of each of these inhibitors and the individual DS variant (at a concentration of 25 µM/mL). Subsequently, the phosphorylation of MLKL was assessed as described above.

### 2.4. Analysis of the DS-Mediated Activation of MLKL by Western Blotting

The BT-474 cells were seeded into a six-well plate (Corning Incorporated, New York, NY, USA) and exposed to PM or NF at a concentration of 25 µg/mL for 3.5 h. After exhaustive rinsing, the cells were lysed in RIPA buffer (40 mM TrisHCl, pH 7.5, 0.15 M NaCl, 0.002 M EDTA, 0.5% Igepal CA630, 0.5% sodium, 0.1% sodium dodecyl sulfate (SDS) (all from Sigma-Aldrich, St. Louis, MO, USA) as well as protease (Mix M, Serva GmbH, Heidelberg, Germany) and phosphatase (Set III, Merck, Rahway, NJ, USA) inhibitor cocktails. The lysates were incubated for 30 min at 4 °C under agitation, and then centrifuged (15,000× *g*, 20 min, 4 °C). The protein concentration was measured in the supernatants obtained using a Pierce BCA Protein Assay Kit (Thermo Fisher Scientific, Rockville, MD, USA). The aliquots containing 15 µg of protein were resolved using sodium dodecyl sulfate polyacrylamide gel electrophoresis (SDS-PAGE) 4–20% MiniProtean TGX gel (Bio-rad, Hercules, CA, USA). Then, the resolved proteins were transferred onto Immobilon P membranes (Sigma-Aldrich, St. Louis, MO, USA) and probed overnight with the monoclonal rabbit anti-phospho-MLKL (S358) antibody (# ab187091, Abcam, Cambridge, UK) that was applied diluted at 1:1000 in TBST buffer (0.05 M TrisHCl buffer, pH 7.4, 0.15 M NaCl and 0.1% Tween 20) also containing 5% (*w*/*v*) Blot Quick Blocker (Millipore Corp., Billerica, MA, USA). Subsequently, after an intense washing, the membranes were treated for 1 h at room temperature with peroxidase-conjugated goat anti-rabbit immunoglobulin G antibodies (# A9169, Merck, Rahway, NJ, USA), diluted at 1:12,000 in a TBST buffer that was supplemented with 5% (*w*/*v*) Blot Quick Blocker. The immunoreactive phospho-MLKL protein was visualized using a 3,3′,5,5′-tetramethylbenzidine substrate (Merck, Rahway, NJ, USA). Before probing with the glyceraldehyde-3-phosphate dehydrogenase (GAPDH) antibodies, the blots were stripped in a solution containing 1.5% glycine, 0.1% SDS, 1% Tween 20 and pH 2.2 for 10 min at room temperature and then re-blocked. Next, the blots were probed with rabbit polyclonal anti-GAPDH antibodies (# 2275, Trevigen, Gaithersburg, MD, USA) that had been diluted at 1:2500 followed by a secondary antibody as was described above.

### 2.5. Analysis of the DS Variant-Promoted Effects on the Nuclear Translocation of NFκB by Immunofluorescence

The cancer cells were cultured under the conditions described above. After the exposure to an individual DS variant for a specified period of time as indicated in Section 3, the cells were fixed and permeabilized as described above. The cellular localization of NFκB was examined using 1.5 µg/mL of rabbit polyclonal anti-NFκB p65 antibodies (# ab16502, Abcam, Cambridge, UK) according to the procedure that was described above for the detection of phospho-MLKL by immunofluorescence.

### 2.6. Analysis of the DS Variant-Mediated Nuclear Translocation of NFκB by Western Blotting

The cells were seeded into a six-well plate (Corning Incorporated, New York, NY, USA) at a density of 89,000 cells per well. After exposure to the examined DS variants at a concentration of 25 µg/mL for 45 min (BT-474 line) or 30 min (T-47D line), the cells were intensively rinsed and treated with 0.05% trypsin for 3 min at 37 °C. The detached cells were scraped and transferred to tubes containing PBS with 10% FBS to neutralize trypsin. Subsequently, the cells were centrifuged (450× *g*, 5 min, room temperature), rinsed with PBS and again centrifuged under the same conditions. Cellular pellets were suspended in 10 mM HEPES buffer, pH 7.9, containing 10 mM KCl, 1 mM EDTA and protease (Mix M, Serva GmbH, Heidelberg, Germany) and phosphatase (Set III, Merck, Rahway, NJ, USA) inhibitor cocktails and allowed to swell on ice for 25 min. Then, the cells were homogenized using a syringe with a narrow-gauge (no. 27) hypodermic needle. The disrupted cells were centrifuged (12,000× *g*, 10 min, 4 °C), and the supernatants were discarded. The obtained pellets that contained a nuclear fraction were then lysed in RIPA buffer, containing 20 mM TrisHCl pH 7.5, 150 mM NaCl, 0.1% SDS, 0.5% Igepal CA630, 0.5% sodium deoxycholate (all from Sigma-Aldrich, St. Louis, MO, USA) and protease inhibitor cocktail (Mix M, Serva GmbH, Heidelberg, Germany) for 30 min, at 4 °C, under agitation. Nuclear proteins were separated by centrifugation (21,000× *g*, 10 min, 4 °C), and, after the measuring of their content by a Pierce BCA Protein Assay Kit (Thermo Fisher Scientific, Rockville, MD, USA), they were resolved by SDS-PAGE as described above. Then, after transfer onto Immobilon P membranes (Sigma-Aldrich, St. Louis, MO, USA), the proteins were probed overnight with the polyclonal rabbit anti-NFκB p65 antibodies (# ab16502, Abcam, Cambridge, UK) at a concentration of 0.5 µg/mL and submitted to a further procedure as described above. Subsequently, the blots were striped and probed with a mouse monoclonal anti-histone 3 antibody (# 309551, Abcam, Cambridge, UK) that had been diluted at 1:1000, followed by a secondary antibody (goat anti-mouse antibodies conjugated with horseradish peroxidase (# A2304, Merck, Rahway, NJ, USA)) at a dilution of 1:80,000.

### 2.7. Estimation of the DS Effect on the Organization of Actin Cytoskeleton

The breast cancer cells were seeded on eight-well glass chamber slides (Cat# 154534, Thermo Fischer Scientific, Rockville, MD, USA) and exposed to the DS variants for the appropriate period of time. Then, after washing three times with PBS, the cells were fixed with 3.7% formaldehyde for 10 min, permeabilized in 0.3% Triton X-100 for 5 min and preincubated with PBS containing 1% BSA for 30 min. The actin cytoskeleton of the cells was stained with an Alexa Fluor 488 phalloidin (Cat# A12379, Thermo Fisher Scientific, Rockville, MD, USA) solution (1.5 units of phalloidin per well) in PBS containing 1% BSA for 20 min at room temperature. Finally, after the nuclei were stained with Hoechst 33342, images of the cells were taken using a Leica DMI 6000B microscope (Leica Microsystems GmbH, Wetzlar, Germany).

### 2.8. Fluorescence Analysis of the Oxidative Stress

The breast cancer cells were seeded on eight-well glass chamber slides (Cat# 154534, Thermo Fischer Scientific, Rockville, MD, USA) and exposed to the DS variants at a concentration of 25 µg/mL for an appropriate period of time as indicated in Section 3. Then, after intense washing three times with PBS, the cells were incubated with a growth medium containing 5 μM of CellROX Orange Reagent (Cat# C10443, Thermo Fischer Scientific, Rockville, MD, USA) for 30 min at 37 °C, followed by Hoechst 33342 staining. Images of the stained cells were captured using a Leica DMI 6000B microscope (Leica Microsystems GmbH, Wetzlar, Germany). In some experiments, before the DS was applied, the cells were first preincubated for 3 h with 6 or 12 µM EHop 016 (Tocris, Bristol, UK) and then exposed to the combined action of EHop 016 and an individual DS variant. The ROS production in these cells was assessed as described above.

### 2.9. Quantitative Analysis of the RIPK3 Expression

The breast cancer cells were seeded into a 24-well plate at a density of 18,000 cells per well and allowed to grow for 48 h as described above. Then, the cells were exposed to an individual DS variant and/or the appropriate inhibitor (cardamonin, EHop 016 or rhosin (Tocris, Bristol, UK)). After this time, the total RNA was isolated using the NucleoZOL reagent (Macherey-Nagel, Allentown, PA, USA) according to the manufacturer’s protocol. The obtained RNA extracts were subjected to DNA-se I in order to remove any contamination with genomic DNA. The integrity of the total RNA was assessed electrophoretically. The mRNA copy number for *RIPK3* was determined using RT-qPCR based on the specific primers (KiCqStart^®^ SYBR^®^ Green Primers, Merck, Rahway, NJ, USA) with the following sequences: 5′AACTTTCAGAAACCAGATGC3′ (forward primer (sense)) and 5′GTTGTATATGTTAACGAGCGG3′ (reverse primer (antisense)) according to the manufacturer’s protocol. The gene expression was analyzed using a CFX96 Touch™ Real-Time PCR Detection System (Bio-Rad Laboratories, Hercules, CA, USA) sequence detector. Negative controls without RNA were included in each run of the RT-qPCR. A melting-curve analysis was performed to confirm the RT-qPCR specificity. The results were analyzed using a Bio-Rad CFX Manager v.3.1 (Hercules, CA, USA). The relative gene expression was obtained after normalization with the endogenous control, a gene for human Tata Binding Protein (TBP)*,* and the difference in the threshold cycle (CT) between the treated and untreated cells was determined using the 2^−∆∆CT^ method. Each of the data points for the mRNA copy numbers is the average of the duplicates on the same analyzed plate.

### 2.10. Statistical Analysis

The data were analyzed using the Statistica 13.3 application (TIBCO Software Inc., Cracow, Poland). The normality of the distribution was verified using the Shapiro–Wilk test, whereas the variance homogeneity was analyzed using the Levene’s test. The data were summarized as (1) the mean ± standard error of the mean (SEM) in cell culture experiments or (2) the mean ± standard deviation (SD) in *RIPK3* expression experiments and in Western blot analysis. The between-group comparisons were assessed based on a one-way ANOVA and the post-hoc Tuckey’s test with *p* ≤ 0.05 as being significant. The between-group differences for the non-parametric data were estimated using the Kruskal–Wallis test by ranks with *p* ≤ 0.05 as being significant.

## 3. Results

### 3.1. DS Affects the Activation of MLKL with Dynamics Depending on Its Structure and the Type of Cancer Cells

We previously showed [4] that when applied at a high concentration (25 μg/mL), some structural variants of DS, i.e., DS from normal human fascia lata (NF) as well as DS from fibrosis affected human palmar fascia (DF) and DS from porcine intestinal mucosa (PM) could, within several hours, induce both the binding to annexin V and the activation of the necroptotic executor MLKL in cells of two luminal breast cancer cell lines. In particular, the variants NF and PM were especially effective in triggering these processes in a primary breast cancer line (BT-483), while DF and PM significantly induced them in the metastatic breast cancer cells T-47D [4]. In the present study, we first tested whether NF and PM, when used at the same concentration, could also rapidly trigger the phosphorylation of MLKL in another primary luminal breast cancer cell line, i.e., BT-474, and then we addressed the precise time course not only of this process but also of the one that was induced by DF or PM in the T-47D line. To investigate this, we used two approaches. First, using immunofluorescence, we examined the level of phospho-MLKL in the cells that had been exposed to these glycans for various time periods, i.e., 2.5, 3.5 and 4.5 h, which were partly based on the previous results [4]. However, we omitted the shortest incubation (2.5 h) of T-47D with the DS variants in the present experiment because we had previously observed the lack of cell response at this time point [4]. Next, we verified the obtained immunofluorescence results concerning the maximum activation of MLKL in the BT-474 using a Western blot analysis. Interestingly, this analysis had previously indicated in the T-47D cells [4] that, when PM was present in the cell cultures for four hours, it increased the level of phosphorylated MLKL by 1.5-fold compared to the control, although, unlike DF, it only slightly affected this modification in an immunofluorescence assay.

**Figure 1:**
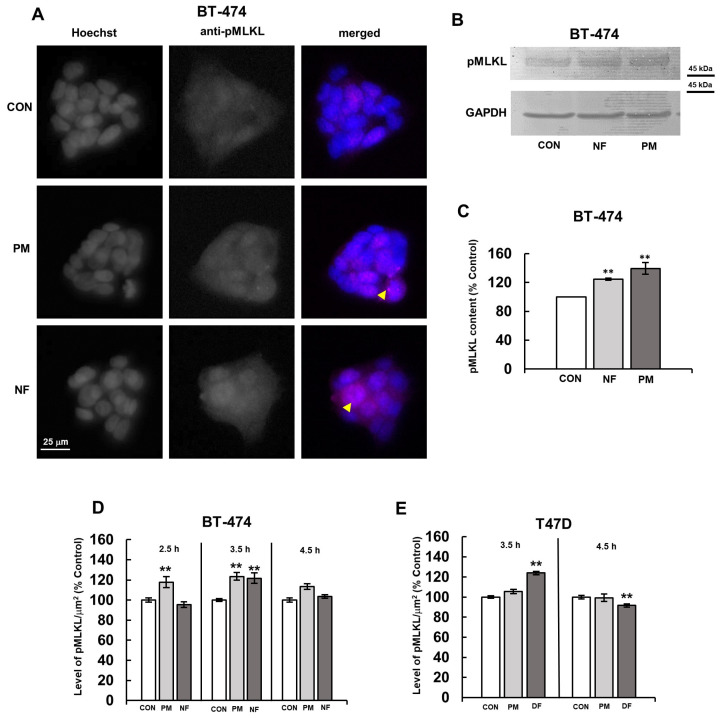
Dermatan sulfate (DS) rapidly induced the activation of the necroptotic executor MLKL in luminal breast cancer cells with dynamics that were dependent on its structure. (**A**) The representative images showing the maximum effect of the structural variants of DS (PM–DS from porcine intestinal mucosa and NF–DS from normal human fascia) on the activation of MLKL in the BT-474 cell line. The cells were exposed to 25 µg/mL of an individual DS variant for 3.5 h. The activated MLKL was detected using the monoclonal antibody against phospho-S358 (Abcam) at a 1:300 dilution; the nuclei were stained with Hoechst. Arrowheads indicate large oligomers of phospho-MLKL. (**B**) The representative Western blotting analysis of the PM- or NF-mediated effect on the level of phospho-MLKL in the BT-474 cells that had been exposed to each of these variants at a concentration of 25 µg/mL for 3.5 h. Then, the cells were lysed in RIPA buffer, and the cellular proteins (15 µg) were submitted to SDS-PAGE. The obtained blots were probed with monoclonal anti-phospho-MLKL (Abcam) at a dilution of 1:1000. Original Western blots can be found in Supplementary Materials. (**C**) Quantitative analysis of the obtained immunoblots, illustrating the maximum DS variant-promoted effect on the activation of MLKL in the BT-474 cells. The levels of phospho-MLKL were normalized to the GAPDH content. The results are expressed as the mean ± SD of three independent experiments. **—statistically significant differences (*p* < 0.01) versus the control. (**D**,**E**) The kinetics of the DS variant-mediated activation of MLKL in the BT-474 (**D**) and T-47D (**E**) cells. The cells were incubated with the DS variants (PM, NF or DF–DS from fibrosis-affected human fascia) for the indicated time periods. The activation of MLKL was estimated as the fluorescence per µm^2^ for all of the visible cells in six non-overlapping fields from each of the five independent experiments. The results are expressed as the percentage of the effect visible in the untreated controls and are presented as the mean ± SEM that was calculated for all of the obtained images. **—statistically significant differences (*p* < 0.01) versus the control.

The obtained immunofluorescence results showed (Figure 1A) that the activation of MLKL in BT-474 manifested itself in two ways: (1) as a fine-grained immunofluorescence that was scattered throughout the cytoplasm of most, if not all, of the cells in compact cell clusters, which was detected in both the control and PM- or NF-treated cells; (2) as large bright punctate structures that were visible at the periphery of single cells in the cell groups, which was mainly detected in the PM- or NF-treated cultures. This appearance was similar to the effect that had been previously observed in the NF-treated BT-483 cells [4].

In order to quantify the effect of the examined DS variants on the activation of MLKL in BT-474 and T-47D, and taking into account the diffuse manifestation of this phenomenon in both cell lines (Figure 1A and [4]), we assessed the average fluorescence per µm^2^ for all of the visible cells in the microscopic preparations. This analysis showed that almost all of the tested variants of DS significantly increased the level of phospho-MLKL in both cancer lines, with the maximum effect being detected after 3.5 h of exposure (Figure 1D,E). Moreover, the ability of both PM and NF to significantly induce the activation of MLKL in the BT-474 cells was also confirmed by the Western blotting analysis (Figure 1 B,C), the results of which allowed us to conclude that the real biological effects of these glycans, especially PM, could be even stronger than indicated by the immunofluorescence data (Figure 1D). However, the kinetics of the MLKL activation in these cells, when it was analyzed using immunofluorescence, varied depending on the DS variant that was used. PM triggered a progressive increase in the level of phospho-MLKL, in which significant changes were already observed after a 2.5 h incubation, and a slow normalization of this parameter at 4.5 h (Figure 1D). In turn, NF caused a sudden stimulation of the MLKL phosphorylation in the BT-474 cells, which was only visible after 3.5 h of exposure (Figure 1D). These data, together with the results of the previous study [4], which used the BT-483 cells, indicate that the DS variants can induce the activation of MLKL in various primary luminal breast cancer lines with the dynamics of the process depending on the cell type and structure of these glycans.

All of the current and previous [4] data clearly showed that an immunofluorescence analysis of the DS variant-induced activation of MLKL in luminal breast cancer cells, although less sensitive than Western blotting, can be used to assess the quantitative effects that are triggered by these glycans. This was important because an immunofluorescence required much smaller amounts of the unique human DS variants that we had to use at a high concentration, compared to Western blotting. Thus, we used this approach to quantify the activation of MLKL in the breast cancer cultures that had been exposed to the tested DS variants for 3.5 h in order to investigate the intracellular events leading to this process.

### 3.2. DS Can Affect the Phosphorylation of MLKL in Breast Cancer Cells via the NFκB Pathway

**Figure 2:**
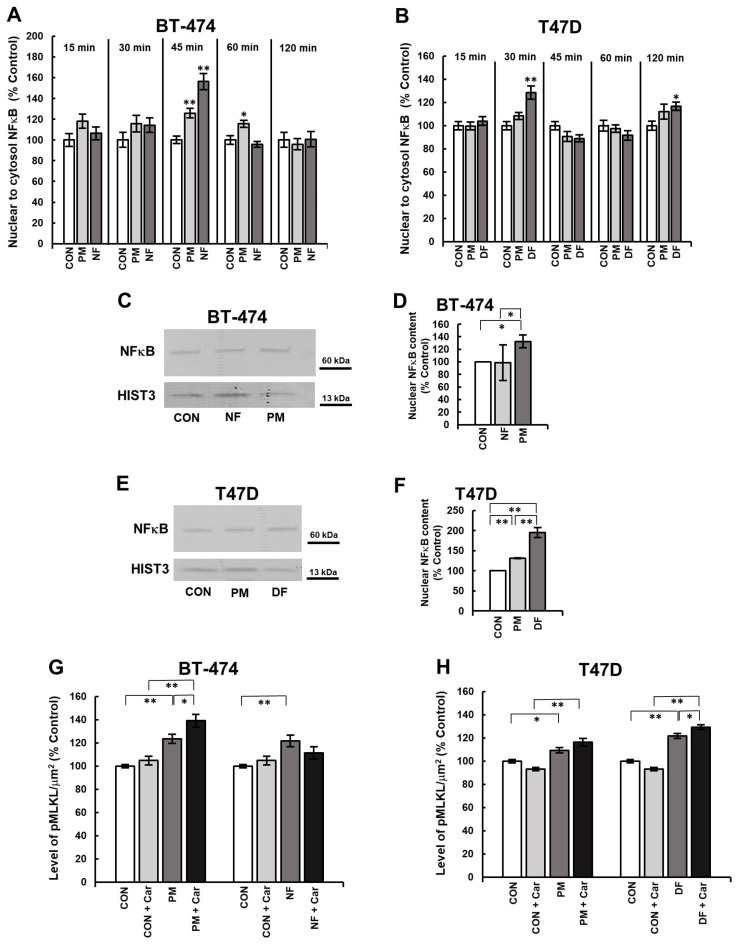
DS stimulated the activation of NFκB and via this process affected the phosphorylation of MLKL in luminal breast cancer cells. (**A**,**B**) the dynamics of the DS variant-mediated activation of NFκB in the BT-474 (**A**) and T-47D (**B**) cells. The cells had been exposed to the DS variants (PM–DS from porcine intestinal mucosa, NF–DS from normal human fascia and DF–DS from fibrosis-affected human fascia) at a concentration of 25 µg/mL for the indicated periods of time. Then, the cellular localization of NFκB was detected by an immunofluorescence using 1.5 µg/mL of polyclonal antibodies against the human p65 unit (Abcam). The activation of NFκB was calculated as the ratio of nuclear to cytoplasmic immunofluorescence for all of the visible cells. The results are presented as the percentage of the effect that was seen in the untreated control and are expressed as the mean ± SEM for the six non-overlapping images that were obtained in three independent experiments. (**C**,**E**) The DS variants significantly increased the nuclear pool of NFκB in the BT-474 (**C**) and T-47D (**E**) cells, as assessed by a Western blotting analysis. The cells had been exposed to the tested DS variants for 45 min (BT-474 line) or 30 min (T-47D line). Then, the cells were homogenized after swelling in hypotonic HEPES buffer, pH 7.9, and the nuclear fraction was separated by centrifugation. The nuclear proteins (2.5 μg) were submitted to SDS-PAGE and detected by immunoblotting with polyclonal anti-human p65 unit antibodies (Abcam) at a concentration of 0.5 µg/mL. Original Western blots can be found in Supplementary Materials. (**D**,**F**) Quantitative analysis of the obtained immunoblots illustrates the maximum ability of the tested DS variants to stimulate the activation of NFκB in BT-474 (**D**) and T-47D (**F**) cells. The levels of nuclear NFκB were normalized to histone H3 content. The results are expressed as the percentage of the effect that was visible in the untreated controls and are presented as the mean ±SD for three independent experiments. */**—statistically significant differences (*p* < 0.05/*p* < 0.01, respectively). (**G**,**H**) An inhibition of NFκB by 20 µM cardamonin (Car) significantly increased the DS-promoted activation of MLKL in the BT-474 (**G**) and T-47D (**H**) cells in a manner that was dependent on the structure of the glycan. The cells were preincubated with Car for 3 h, and then exposed to a combination of the inhibitor and individual DS variant for 3.5 h. The activation of MLKL was assessed by immunofluorescence. The results are expressed as the percentage of the effect that was visible in the untreated controls and are presented as the mean ± SEM for all of the images that were obtained in three independent experiments. */**—statistically significant differences (*p* < 0.05/*p* < 0.01, respectively).

The level of MLKL activation that is caused by an individual DS variant may depend on this glycan-mediated effect on the pro-survival NFκB pathway. To explore this issue, we first tested the dynamics of the nuclear translocation of NFκB in the exposed cells (Supplementary Figure S1) by measuring the ratio of nuclear to cytosol immunofluorescence at several time points within a 2 h time period in accordance with the canonical model of the stimulation of this transcription factor by TNF α [16]. During this incubation period, both NF and PM significantly increased the NFκB activation in the BT-474 cells compared to the control, and this effect was manifested as a single-wave nuclear translocation of this transcription factor, although with different kinetics (Figure 2A). Unlike NF, which caused a rapid but short-lived nuclear influx of NFκB at 45 min, the PM-induced translocation of this transcription factor was more prolonged, reaching a maximum at 45 min and still remaining at an elevated level after 60 min of incubation (Figure 2A). However, unexpectedly, the Western blot analysis of the nuclear fraction from BT-474 cells that had been exposed to each of these variants for 45 min clearly showed that only PM could significantly increase the nuclear pool of NFκB (Figure 2C,D).

Similar to the BT-474 line, the activation of NFκB in the T-47D cells was also significantly modified by the DS variants in a manner that was dependent on their structure (Figure 2B). The dynamics of the DF-mediated effect was clearly biphasic or oscillatory with the first wave of the nuclear translocation of NFκB occurring at 30 min and a second smaller wave that was manifested at 120 min of the incubation period, which were separated by a slight decrease in the nuclear content of this transcription factor (Figure 2B). In turn, during the observation period, PM stimulated only minor alterations in the nuclear influx of NFκB that reached the maximum level at 120 min of the incubation period (Figure 2B). However, unexpectedly, the Western blot analysis elicited that not only the T-47D cells that were exposed to DF for 30 min but also those growing for the same time period in the presence of PM had a significantly higher nuclear content of NFκB compared to the control (Figure 2E,F).

To investigate whether the DS variant-promoted activation of NFκB could affect the phosphorylation of MLKL that was induced by these glycans in breast cancer cells, we used immunofluorescence to determine the level of phospho-MLKL in cultures that had first been preincubated for 3 h with cardamonin, which is an inhibitor of this transcription factor [17,18,19], and then exposed to cardamonin in combination with an individual DS variant for 3.5 h (Supplementary Figure S2). When it was applied alone, cardamonin had no effect on the content of phospho-MLKL in any of the cell lines that were used (Figure 2G,H). However, it should be emphasized that, especially in the T-47D cells that had been treated with this inhibitor, there was a clear shift in the manifestation of immunofluorescence from fine-grained to large dot-like forms (Supplementary Figure S2), which suggests that the necroptosis induction might be an alternative mechanism for delivering pro-inflammatory cytokines after the NFκB pathway has been blocked. The combined administration of cardamonin and PM triggered an increase in the level of phospho-MLKL in both breast cancer lines compared not only to the corresponding control but also to the parallel cultures that had been treated with the variant alone (Figure 2G,H). This latter comparison allowed us to estimate the specific impact of the PM-mediated activation of NFκB on the phosphorylation of MLKL in both cell lines. As results from Figure 2G,H, cardamonin led to a statistically significant increase in the level of phospho-MLKL only in the BT-474 cells that were treated with this variant. Similar effects were observed when DF was applied together with cardamonin in the T-47D cultures (Figure 2H). In contrast, the combined application of NF and cardamonin did not improve the ability of this variant to activate MLKL in the BT-474 cells (Figure 2G). Therefore, all of the above-presented results indicate that the DS variant-mediated activation of NFκB affects the ability of these glycans to induce the MLKL phosphorylation in luminal breast cancer cells in a manner that is dependent on the structure of these molecules and the cancer cell type.

### 3.3. DS Variants Can Promote MLKL Activation via Their Effects on the Organization of the Actin Cytoskeleton in Breast Cancer Cells

Before examining the involvement of the actin cytoskeleton-associated signaling in the DS-mediated activation of MLKL, we investigated the organization of the actin filaments in breast cancer cells that had been exposed to the tested variants of this glycan during a short incubation (maximum up to 45 min based on the results of Tufvesson and Westerngren-Thornsonn [14]), using Alexa-Fluor-modified phalloidin.

**Figure 3:**
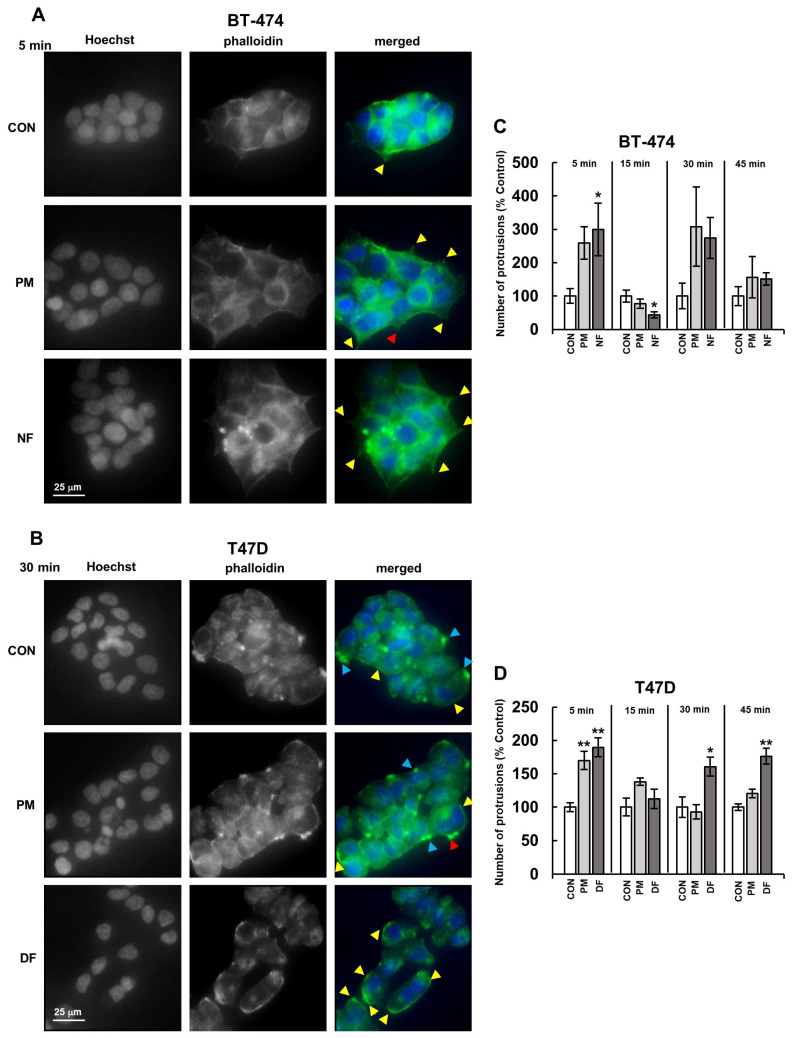
DS rapidly influenced the actin cytoskeleton organization in luminal breast cancer cells. (**A**,**B**) Representative images showing the effects of the DS variants (PM–DS from porcine intestinal mucosa, NF–DS from normal human fascia, DF–DS from fibrosis-affected human fascia) at a concentration of 25 µg/mL on the formation of cellular protrusions in the BT-474 (**A**) or T-47D (**B**) cells that were incubated with the glycans for the indicated time periods and then stained with phalloidin-Alexa Fluor (actin filaments–green) and Hoechst (nuclei–blue). Yellow arrowheads (**A**,**B**) indicate lamellipodia-like processes, blue arrowheads (**B**)–large dot-like structures, and the red arrowhead (**A**,**B**) shows clusters of projections resembling filopodia. (**C**,**D**) The dynamics of the DS variant-mediated effects on the formation of lamellipodia-like protrusions in the BT-474 (**C**) and T-47D (**D**) cells. The cells were grown in the presence of the DS variants for the indicated periods of time. The number of protrusions (indicated by yellow or blue arrowheads in the BT-474 or T-47D cells, respectively) was counted for all of the visible cells in four non-overlapping fields from each of two independent experiments. The results are expressed as the percentage of the effect that was visible in the untreated controls and are presented as the mean ±SEM for all of the obtained images. */**—statistically significant differences (*p* < 0.05/*p* < 0.01, respectively) versus the control.

The cytoskeleton organization in the cultured luminal breast cancer cells had a cell-type-specific character. The BT-474 line grew in clusters containing tightly adhered cells that exhibited several characteristic features of the actin cytoskeleton organization (Figure 3A). These included (1) the presence of short, pointed protrusions with centrally accumulated actin filaments in the cells lying at the periphery of the clusters, (2) the assembly of actin into the cortical layer under the plasma membrane, as well as (3) a small number of broad lamellipodia-like protrusions that contained dot-like structures (resembling fibrillar complexes) and that often coexisted with small clusters of short, thin finger-like projections (probably representing filopodia) (Figure 3A). In contrast, T-47D cells, which grew in more loose groups, had actin filaments that were mainly assembled into a pronounced layer and large bright dot-like structures within flat, wide projections that resembled lamellipodia (Figure 3B). Moreover, small clumps of short, finger-like protrusions (possibly filopodia) and a single large, point-like structure, which were located on the cell bodies, were also observed (Figure 3B).

**Figure 4:**
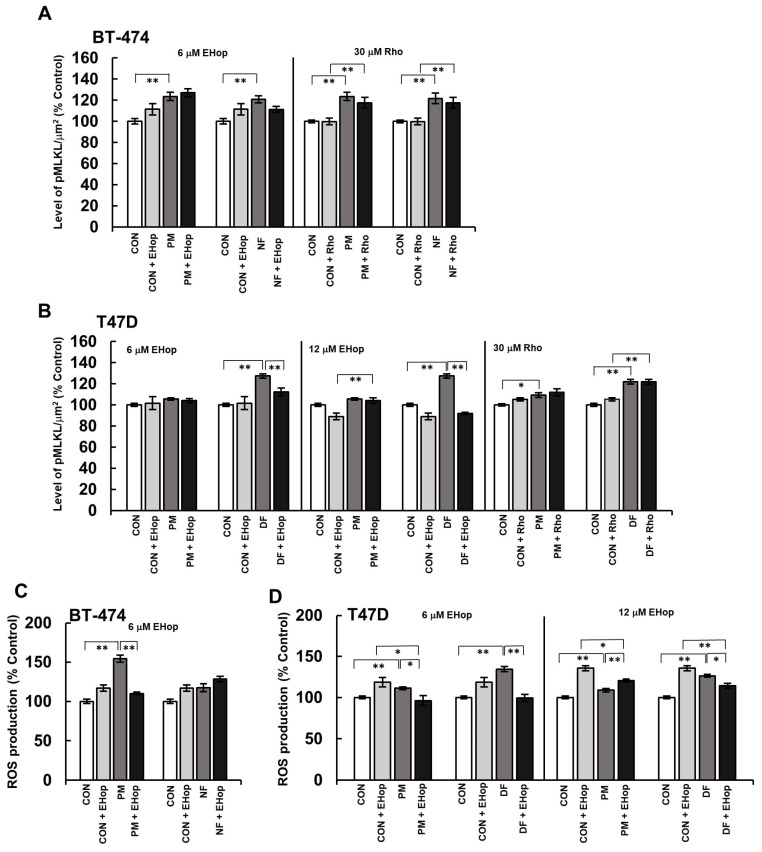
DS via its effect on the actin cytoskeleton rearrangement induced the phosphorylation of MLKL as well as the redox imbalance in luminal breast cancer cells. (**A**,**B**) The pharmacological inhibition of small Rho GTP-ases affected the ability of the DS variants (PM–DS from porcine intestinal mucosa, NF–DS from normal human fascia, DF–DS from fibrosis-affected human fascia) to activate MLKL in the BT-474 (**A**) and T-47D (**B**) cells. The cells were preincubated with the indicated concentrations of Rac1/Cdc42 inhibitor (EHop 016) or RhoA inhibitor (rhosin, Rho) for 3 h. Then, the cells were exposed to the combined action of the inhibitor and an individual DS variant for 3.5 h. The activation of MLKL was assessed by immunofluorescence. (**C**,**D**) The DS-induced oxidative stress was preceded by this glycan-mediated effect on the Rac1 activity in the BT-474 (**C**) and T-47D (**D**) cells. The ROS production was measured using Cell ROX Orange in the breast cancer cells that were first treated with the indicated concentrations of EHop 016 for 3 h, and then submitted to the combined action of an individual DS variant and EHop 016 for 45 min. The results are expressed as the percentage of the fluorescence that was detected in the untreated controls and are presented as the mean ± SEM for all of the obtained images in three independent experiments. */**—statistically significant differences (*p* < 0.05/*p* < 0.01, respectively).

Our data (Figure 3C,D) clearly indicated that all of the tested DS variants apparently and quickly influenced the actin cytoskeleton morphology primarily in a cell-type-dependent manner. The most prominent alteration in the actin filament organization of the exposed cells was a significant increase in the number of cell-line-specific projections (Figure 3A–D). Interestingly, this effect was clearly biphasic or oscillatory, manifesting at 5 and 30 min of the incubation period, especially in the BT-474 cells (Figure 3C) but also in the T-47D cells that had been treated with DF (Figure 3D). In contrast, when it was used in the T-47D line, PM stimulated the formation of cellular protrusions only after the 5 min incubation period (Figure 3D). In addition to the above-mentioned changes in the actin cytoskeleton organization, other DS-promoted effects were also visible, including a marked increase in the number of finger-like protrusions (filopodia) in the T-47D cells that had been incubated with PM for 30 min (Figure 3B). Furthermore, in the BT-474 cells that had been exposed specifically to NF, the cortical actin layer was more condensed than in the other cultures (Figure 3A). To summarize, the obtained results clearly indicated that the tested DS variants could rapidly influence the actin cytoskeleton organization in breast cancer cells, which suggests the impact of these glycans on the activity of at least two small Rho GTP-ases, i.e., Rac1 and Cdc42, which are responsible for the formation of the lamellipodia and filopodia, respectively [15]. As such events can lead to alterations in the intracellular signaling, thereby inducing pro-necroptotic stimuli [6,7], we examined whether the inhibition of the activity of the small GTP-ases by pharmacological agents could affect the DS variant-mediated phosphorylation of MLKL (Supplementary Figure S3). For this purpose, two compounds were used—EHop 016, which at low concentrations (<10 µM) can exclusively inhibit the Rac1 activity, while at higher concentrations, it also blocks Cdc42 [20], and rhosin, which is an inhibitor of RhoA [21]. When used alone, neither of these inhibitors significantly affected the level of phospho-MLKL in either cancer cell lines compared to the untreated controls (Figure 4A,B). However, in the presence of 6 µM EHop 016, the differences in the level of phospho-MLKL between the DS variant-treated and the control BT-474 cells were at least reduced (PM) or even abolished (NF) (Figure 4A), thus indicating the involvement of Rac1 in the activation of this necroptotic effector. Moreover, at a concentration of 6 µM, EHop 016 significantly limited the ability of DF to trigger the phosphorylation of MLKL in the T-47D cells, while it had no impact on the action of PM in these cells (Figure 4B). On the other hand, when EHop 016 was applied to the BT-474 cultures at 12 µM, it significantly reduced the number of these cells, which made it impossible to conduct the experiment. This observation clearly indicates that maintaining the activity of at least one of the small Rho GTP-ases, which are inhibited by EHop 016, i.e., Rac1 or Cdc-42, is crucial for the survival of BT-474, whereas the viability of the T-47D cells was less sensitive to the inhibition of both of these enzymes. The application of EHop 016 to these cells at 12 µM completely abolished the DF-induced activation of MLKL, thus suggesting a key role of Cdc-42 along with Rac1 in this process (Figure 4B). In contrast, unexpectedly, EHop 016 at 12 µM revealed the ability of PM to induce the phosphorylation of MLKL in the T-47D cells, which suggests that the variant could trigger activities that oppose this process via the Rac1/Cdc42 pathway (Figure 4B).

In contrast to EHop 016, rhosin actually had no impact on the activation of the MLKL that was triggered by the tested DS variants (Figure 4A,B). In conclusion, the above results clearly indicate that DS can not only initiate changes in the actin filament organization in luminal breast cancer cells, but, via the signaling that is associated with this process, this glycan can induce pro-necroptotic stimuli in a manner that is dependent on its structure and tumor cell type.

### 3.4. DS Influences the Activation of MLKL via the Induction of Oxidative Stress in Breast Cancer Cells

**Figure 5:**
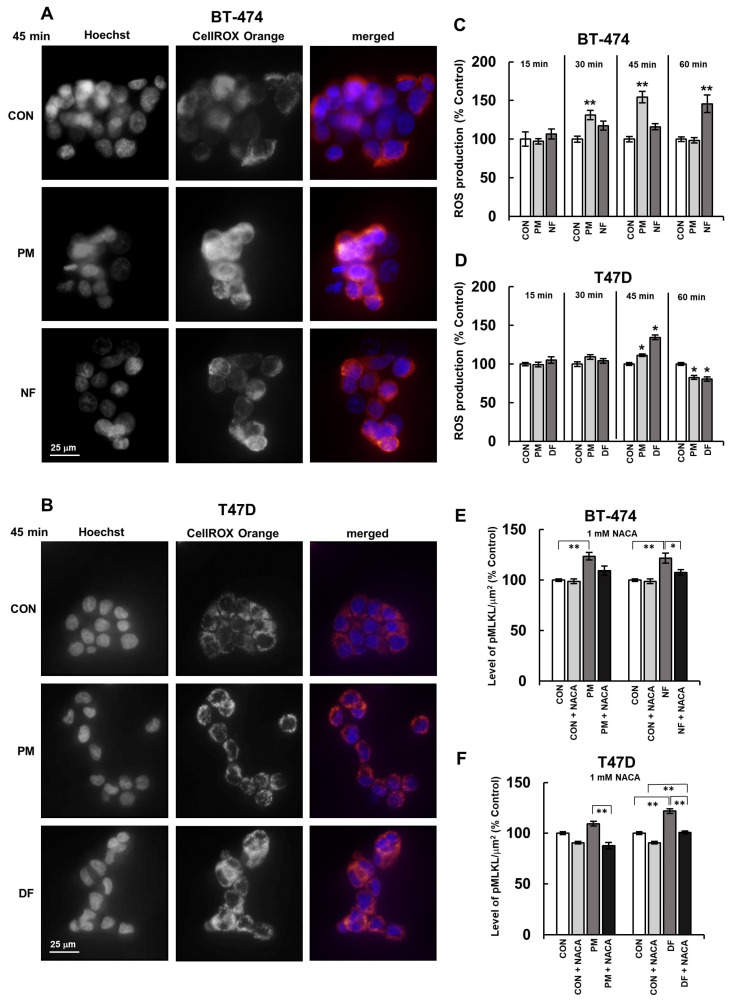
DS rapidly triggered the redox imbalance and, via this mechanism, induced the activation of MLKL in luminal breast cancer cells. (**A**,**B**) Representative images showing ROS production in the BT-474 (**A**) and T-47D (**B**) cultures that were exposed to the DS variants (PM–DS from porcine intestinal mucosa and DF–DS from fibrosis-affected human fascia) for the indicated period of time. Then, the cells were stained with Cell ROX Orange (ROS production) and Hoechst (nuclei, blue). (**C**,**D**) The dynamics of the DS variant-promoted induction of short-term oxidative stress in the BT-474 (**C**) and T-47D (**D**) cells. The cells were exposed to an individual DS variant at a concentration of 25 µM/mL for the indicated periods of time and then treated with Cell ROX Orange. (**E**,**F**) The DS-mediated activation of MLKL in the BT-474 (**E**) and T-47D (**F**) cells was dependent on this glycan-induced oxidative stress. The activation of MLKL was measured by immunofluorescence in the breast cancer cells that were first preincubated with 1 mM of N-acetylcysteine amide (NACA) for 3 h and then subjected to a combined treatment with an individual DS variant and NACA for 3.5 h. The fluorescence per µm^2^ was taken from all of the visible cells in six non-overlapping fields from each of the three independent experiments. The results are expressed as the percentage of the fluorescence that was detected in the untreated controls and are presented as the mean ± SEM for all of the obtained images. */**—statistically significant differences (*p* < 0.05/*p* < 0.01, respectively) versus the control.

We previously found [4] that the structural variants of DS, when they were used at a concentration of 25 µg/mL, could induce oxidative stress in the BT-483 and T-47D breast cancer cells. In the present study, we explored whether these compounds might also trigger the redox imbalance in the BT-474 line. Moreover, we precisely examined the short-term dynamics of this DS-mediated effect not only in the BT-474 line but also in the T-47D line using the fluorescence sensors of the oxidation reaction. The results obtained (Figure 5A–D) together with our previous data [4] suggest that DS can affect the oxidative-reductive balance in various luminal breast cancer cells. However, the kinetics of this effect seems to strongly depend on both the glycan structure and/or the cell type. In the BT-474 cells, both of the tested DS variants induced oxidative stress of a similar maximum intensity but with different dynamics (Figure 5C). The PM-mediated effect occurred faster and lasted longer than that of NF, which was manifested as significant changes in the ROS production that were visible as early as at 30 min of the incubation with the former variant, and persisted for the next 15 min (Figure 5C). In contrast, NF induced oxidative stress in the BT-474 cells only at 60 min of their incubation (Figure 5C). In turn, DF and PM triggered a redox imbalance with similar dynamics but with different intensities in the T-47D cells (Figure 5D). Indeed, both variants showed the maximum stimulatory impact on the ROS production at 45 min of incubation, but the DF-promoted effect was more pronounced than the one that was induced by PM (Figure 5D). However, the redox imbalance that was rapidly induced by the DS variants in the T-47D cells was transient and was replaced by a marked decrease in the ROS production (Figure 5D). This suggests that the mechanism(s) that are involved in the DS-generated oxidative stress might rapidly dissipate or that the tested variants might rapidly release an antioxidant response in exposed cells.

In order to explore the involvement of the DS variant-mediated oxidative stress in the activation of MLKL by these molecules, we used immunofluorescence to measure the level of phospho-MLKL in the breast cancer cells that were exposed to these glycans with or without NACA, which is a universal antioxidant agent [22] (Supplementary Figure S4). NACA alone did not affect the phosphorylation of MLKL in the BT-474 cells but had a slightly reducing effect on this process in the T-47D cultures (Figure 5E,F). In turn, the presence of NACA in the BT-474 cultures not only markedly decreased the differences in the levels of phospho-MLKL between the DS variant-treated and control cells but also significantly reduced the ability of NF to induce the activation of this necroptotic effector (Figure 5E). Moreover, NACA significantly reduced both the DF- and PM-mediated phosphorylation of MLKL in the T-47D cells (Figure 5F).

All of these data clearly indicate that the DS variants promote their activating effect on MLKL by inducing transient oxidative stress in luminal breast cancer cells. Intriguingly, the inhibitory effects that NACA and EHop 016 exhibited on the phosphorylation of MLKL, which was caused by at least some DS variants, were quite similar in their extent (Figure 5E,F vs. Figure 4A,B). This finding suggests that the DS-induced changes in the redox balance and in the Rac1/Cdc42 activity might function interactively via the same pathway in an upstream–downstream cooperative manner leading to the activation of MLKL. However, since the occurrence of changes in the organization of the actin cytoskeleton temporally preceded the induction of oxidative stress (Figure 3C,D vs. Figure 5C,D), we assumed that the DS-triggered alterations in the activity of some small Rho GTP-ases might be the event(s) that led to the redox imbalance. Therefore, in order to verify this hypothesis, we examined the oxidative status of breast cancer cells that were first preincubated for 3 h with the appropriate concentration of EHop 016 and then exposed to this compound in combination with an individual DS variant for 45 min (Supplementary Figure S5). The time conditions that were chosen for this experiment corresponded to the time period that ensured that the maximum effect of most of the variants that were used to induce oxidative stress would be observed.

Notably, at a concentration of 6 µM, EHop 016 alone was able to induce mild oxidative stress in both cancer lines, and this effect was even more pronounced when the inhibitor was applied to the T-47D cells at 12 µM (Figure 4C,D). These data suggest that Rac1 and/or Cdc42 participate in controlling the redox balance in both breast cancer cell lines. However, the inhibition of Rac1 prior to the application of NF, but especially PM in the BT-474 cultures, completely protected the cells from a further increase in oxidative stress, resulting from the action of these variants, which was expected (Figure 4C). Similarly, the ability of the tested DS variants, especially DF, to induce the redox imbalance in the T-47D cells was completely abolished after the Rac1 activity was blocked, and this effect was also maintained after the inhibition of both small GTP-ases (Figure 4D). Thus, these data support the possibility that the DS variant-mediated changes in the activity of Rac1 and Cdc42 are upstream of inducing oxidative stress by these glycans in luminal breast cancer cells.

### 3.5. The DS Variants Can Affect the Activation of MLKL via Their Impact on the RIPK3 Expression

**Figure 6:**
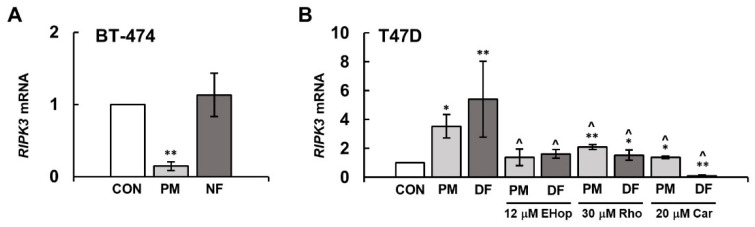
DS influenced the expression of *RIPK3* in luminal breast cancer cells, and this effect was mediated via NFκB and small Rho GTP-ases at least in the T-47D cells. The level of *RIPK3* mRNA was quantified using RT-qPCR in the cultures of BT-474 (**A**) and T-47D (**B**), which were treated with the tested variants of DS (PM–DS from porcine intestinal mucosa, NF–DS from normal human fascia and DF–DS from fibrosis-affected human fascia) at a concentration of 25 µg/mL and/or the indicated concentration of cardamonin (Car), EHop 016 or rhosin (Rho) for 3 h. Control values were normalized to the endogenous human gene for Tata Binding Protein. The results show relative *RIPK3* mRNA expression and are presented as the mean ± SD of four independent experiments. */**—statistically significant differences (*p* < 0.05/*p* < 0.01, respectively) versus the control. ^—statistically significant differences (*p* < 0.001) versus an appropriate DS variant.

As the activation of MLKL is associated with its phosphorylation by RIPK3 [5], the effect of DS variants on the phospho-MLKL level in breast cancer cells might probably be mediated through the stimulatory impact of these glycans on the kinase activity. However, the tested DS variants might also affect the cellular level of RIPK3, regulating the expression of its gene. To investigate this possibility, we measured the content of *RIPK3* mRNA in the breast cancer cells that had been exposed to the tested DS variants for 3 h using real time qPCR. The observed effects depended not only on the structure of the DS variants but mainly on the cancer cell type (Figure 6A,B). In the BT-474 cells, none of the variants that were used increased the content of *RIPK3* mRNA, and PM even exhibited a strongly reducing effect on this parameter (Figure 6A). In contrast, both DS variants that were applied to the T-47D line, but especially DF, significantly increased the content of the *RIPK3* mRNA levels in the exposed cells compared to the untreated cultures (Figure 6B).

To further test which cellular pathways in T-47D might be involved in the DS variant-promoted stimulation of the *RIPK3* expression, we measured the mRNA level for this kinase in the cells that had been incubated with cardamonin, rhosin or EHop 016 for 3 h together with the individual glycan. Each of these inhibitors, when present in the T-47D cultures that had been exposed to PM, either almost completely abrogated (EHop 016) or markedly reduced (rhosin and cardamonin) the stimulatory impact of the variant on the *RIPK3* expression (Figure 6B). Such a mode of inhibitory action suggests that small Rho GTP-ases and NFκB might cooperate within the same PM-induced pathway, thereby leading to the stimulation of the *RIPK3* expression. In contrast to the inhibitors of small GTP-ases, which abolished the stimulatory effect of DF on the content of *RIPK3* mRNA, cardamonin almost completely blocked the gene expression in the DF-exposed cells (Figure 6B). These results indicate that DF can trigger additional signals via NFκB that strongly stimulate the expression of *RIPK3*.

## 4. Discussion

DS is an important component not only of the extracellular matrix in normal tissues, but it also accumulates in the tumor microenvironment. Our present examinations together with our previous results [4] indicated that at least certain structural variants of DS can significantly stimulate the activation of the necroptotic effector MLKL in various luminal breast cancer cell lines, including both the primary tumor and metastatic cells. Moreover, the mode of the manifestation of this DS-dependent activation is also similar in different luminal breast cancer cells, which is reflected in the co-occurrence of two types of structures containing MLKL phosphorylated at S358: the more common fine-grained structures and the rarer large aggregates. Only the level of this latter form of activated MLKL correlated with the number of dying, annexin-positive cancer cells in the previous study [4], which suggests that such structures represent the phospho-MLKL oligomers that are responsible for the execution of necroptosis. In turn, the role of the fine-grained phospho-MLKL in luminal breast cancer cells remains unclear. However, it is known that the activated MLKL can also perform other non-necroptotic functions such as participating in endosomal trafficking and receptor recycling or the ESCRT (Endosomal Sorting Complexes Required for Transport)-dependent repair of the plasma membrane and release of extracellular vesicles as well as inhibiting autophagic flux or affecting gene induction (for review: [23]).

In the present study, we used previously tested structural variants of DS to investigate the mechanism(s) that are implicated in this glycan-promoted phosphorylation of MLKL. The variants tested clearly differed in their epimerization and sulfation patterns [4]. In contrast to PM, which had the highest content of both disaccharides with iduronate residue and 4-O-sulfated disaccharides and was almost devoid of unsulfated disaccharides, NF and especially DF were characterized not only by higher levels of unsulfated disaccharides but also by a markedly higher proportion of hybrid structure, i.e., CS/DS in their chains [4]. The current investigation using these variants clearly indicates that the two intracellular events that are triggered by them in luminal breast cancer cells are principally responsible for the ability of these molecules to induce MLKL phosphorylation. The first of these processes, i.e., the activation of NFκB exerts a suppressive effect, whereas oxidative stress has a stimulatory impact as evidenced by the influence of the pharmacological inhibition of these processes on the MLKL phosphorylation that was promoted by the DS variants. Moreover, at least in the metastatic T-47D cancer line, the DS variants also increased the expression of *RIPK3* via the pathways that require the activity of the small Rho GTP-ases and that are also regulated by NFκB.

Many breast cancer cells exhibit a constitutively increased activation of NFκB [16]. This transcription factor plays a key role at every stage of tumorigenesis by stimulating the proliferation and survival of cells in the cancer microenvironment via the induction of inflammation and the promotion of angiogenesis as well as facilitating metastasis and triggering a resistance to treatment [24,25]. To the best of our knowledge, the impact of DS on the activity of NFκB in cancer cells has not previously been examined. Our study shows that the dynamics of this process clearly depends on the structure of DS and the type of cancer cells. NF and DF induced the activation of NFκB in the form of one or two short-lived waves of the nuclear relocation of this factor, respectively, during the observation period. Interestingly, the activating effect that was exerted by these variants, sharing the above-mentioned features of their sulfation and glucuronosyl epimerization patterns with DS from the tumor niche, was most potent in the more aggressive breast cancer cells, i.e., T-47D. In contrast, PM, which was characterized by a structure typical of DS from normal tissues, caused a rather slowly increasing stimulation of NFκB activation that was more pronounced in the less aggressive breast cancer cells, i.e., BT-474, compared to NF- or DF-promoted effect. This relationship between the DS structure and its effect on the NFκB activation was further reflected in the observed pronounced impact of the pharmacological inhibition of this process on the PM- and DF-induced activations of MLKL in BT-474 and T-47D, respectively. In addition, different patterns of the nuclear translocation of NFκB, which were triggered by PM and DF in the T-47D cells, might contribute to the observed distinct effects of these variants on the NFκB-dependent expression of *RIPK3*. On the other hand, the relationship between the structure of DS and its impact on NFκB activation, which we found in breast cancer cells differing in their aggressiveness, points to the importance of the structural remodeling of this glycan, which is observed in the cancer microenvironment, to generate stimuli that support the survival of cancer cells at different stages of tumor development. Interestingly, a similar relationship between the structure of the DS variant and its biological effect in the breast cancer lines with various degrees of aggressiveness, as observed in the case of NFκB activation, was also noted regarding the influence of the tested glycans on the actin cytoskeleton rearrangement and oxidative stress induction. However, it is unclear whether all of these effects are triggered via interactions of the tested DS variants with a single type of cell surface receptor or by completely separate mechanisms. In addition to the different responsiveness of BT-474 and T-47D to the structurally different DS variants, we also observed marked differences in both the dynamics and intensity of the effects that were induced by PM in both cell lines. These differences might result from several possible reasons including the following: (1) the distinct types of cell surface receptors that were involved in the interactions with this DS and (2) variations in the cellular metabolism, signaling pathways or cell cycle kinetics between the BT-474 and T-47D cells.

Cellular ROS not only accomplish detrimental functions, but they are also an important element in intracellular signaling pathways, which are especially involved in the growth factor action or adhesion [26]. Moreover, many of the proteins that are engaged in signaling are redox sensors [26]. Therefore, it was not unexpected that increased ROS production was also involved in the necroptotic events. However, the location of the oxidative stress in the pathway that leads to necroptosis can be different, depending on the cell type and also perhaps on the kind of stimulus that is triggering this death. In neutrophils, in which necroptosis was induced by the ligation of adhesion molecules following GM-CSF priming, oxidative stress was downstream of the activation of p38 MAPK and PI3K, which had been preceded by phosphorylation of RIPK3 and MLKL [7]. However, it should be emphasized that the overproduction of ROS was necessary for necroptosis to occur in this cellular model [7]. In contrast, the overproduction of mitochondrial ROS, which is essential for TNFα-induced necroptosis in several types of cells, led to the oxidation of RIPK1 followed by its autophosphorylation at serine161, which subsequently triggered the activation of RIPK3 first and then MLKL and the induction of necroptosis [9]. Our data clearly indicate that the DS variant-induced redox imbalance is an event upstream of the MLKL activation that was promoted by these molecules in luminal breast cancer cells, although the involvement of oxidized RIPK1 in this process remains unknown. However, the significant but incomplete sensitivity of the DS-promoted MLKL phosphorylation to the universal ROS scavenger NACA in both tested breast cancer cell lines enabled us to conclude that the oxidative stress that is induced by this glycan is only the main, but not the only, event that is involved in the induction of necroptosis in these cells. Furthermore, our results clearly show that the triggering of the redox imbalance by the DS variants occurs downstream of the modulatory effect of these glycans on the activity of Rho GTP-ases, especially Rac1. This results from the fact that the pharmacological inhibition of this enzyme is already sufficient to completely abolish the DS-dependent ROS production in the exposed cells. In addition, there is a visible correlation between the impact of the tested variants on the formation of lamellipodia-like projections, which is under the control of Rac1, and the ability of these glycans to induce oxidative stress in breast cancer cells.

Rho GTP-ases, including Rac1, have a wide range of functions beyond their influence on the organization of the actin cytoskeleton [27]. In both phagocytic and non-phagocytic cells, it has been shown that Rac1 is required for the activity of NADPH oxidase as an activator of its NOX subunit [28]. Thus, such a mechanism could explain the involvement of Rac1 in the DS-dependent induction of oxidative stress in luminal breast cancer cells. Interestingly, mitochondrion has been suggested as a source of ROS in the Rac-promoted stimulation of oxidative stress in rabbit fibroblasts [29]. However, our previous report showed that the dynamics of the short-term effects of the DS variants on the mitochondrial membrane potential did not correlate with both occurrences of the redox imbalance and necroptosis induction that were triggered by these molecules in the luminal breast cancer cells [4]. Hence, it is possible that the DS variant-triggered ROS overproduction recruits Rac1-dependent oxidase(s) with a different cellular localization, e.g., those that are present in the plasma/endosomal membrane.

Previous reports have shown that in contrast to Rac1, Cdc42 does not directly activate NADPH oxidase [30]. However, only a combined overexpression of constitutively active Rac1 and Cdc42 significantly stimulates superoxide anion production in cardiomyocytes [31]. In turn, our data suggest that the role of Rac1 and Cdc42 in controlling the redox balance in breast cancer cells might be complex. In addition to the involvement of the first enzyme in the DS-dependent induction of oxidative stress, both of these small GTP-ases rather reduce the ROS production at least in the T-47D cells. Such a conclusion can be drawn from the observed consequences of the pharmacological inhibition of these enzymes on the oxidative stress in the cancer cells. However, this finding requires further investigation. Nevertheless, Cdc42 (together with Rac1) plays a role at least in the DF variant-promoted activation of MLKL in the T-47D breast cancer cells. This is supported by our observation that only a simultaneous inhibition of both these small GTP-ases results in the complete suppression of this glycan-dependent process.

The ox-LDL-mediated overproduction of ROS in mesangial cells, which is dependent on the Rac1 activity, requires the activation of β4 integrin [32]. Moreover, ligation, i.e., the activation after binding to the ECM ligand of the α5 integrin led to a Rac1-promoted increase in the ROS production in rabbit synovial fibroblasts [29]. As all of the tested DS variants caused not only a Rac1-dependent induction of oxidative stress in the exposed breast cancer cells but also the rearrangement of the actin cytoskeleton, both of which are initiated by alterations in the integrin activity, we assume that these glycans could affect the function of those adhesion receptors at the surface of cancer cells. It has been shown that DS can directly influence the activity of β1 integrin most probably via a reduction of pH in the pericellular space [33]. Furthermore, CS, which structurally is closely related to DS, especially the type that is termed oncofetal, can directly interact with integrins α4, α5β1 and β1 and affect integrin-mediated signaling [34]. Moreover, both CS and DS can modulate the expression of some integrins on both the mRNA and protein levels in a manner that is dependent on the amount or structure of these glycans [35,36]. Thus, based on the observed differences between the DS variants, not only in terms of their effects on the processes requiring integrin activity but also taking into account the observed specific phase course of these glycan-induced rearrangements of actin cytoskeleton, it is tempting to speculate that the tested molecules might not only exhibit a different integrin binding profile, but they might also actively affect the expression of these adhesion receptors on the surface of luminal breast cancer cells. However, the observed DS variant-triggered alterations in the actin cytoskeleton of breast cancer cells may also result from the interaction of these glycans with CD44, which is an another adhesion molecule. DS has been reported to be a binding partner for this cell surface molecule [37], which is not only capable of indirectly linking with actin filaments via interactions of its intracellular domain with ezrin/radixin/moesin, as well as ankyrin, but also can affect the activity of small Rho GTP-ases including Rac1 (for review [38]). In concluding, it seems that the effect on adhesion receptors might play a fundamental role in the activation of MLKL and the induction of necroptosis that were triggered by high doses of DS in luminal breast cancer cells. Furthermore, the observed biological effect of DS on cancer cells suggests a certain application potential of this glycan in anticancer treatments because the induction of necroptosis in the tumor microenvironment may at least disturb, if not abolish, the immunological tolerance of the host towards transformed cells, which appear during the progression of the disease and constitute a significant therapeutic problem. However, further studies should be undertaken to elucidate the detailed mechanism of DS action and, especially, the relationship between the structure of this glycan and its biological properties.

In the present study, we almost exclusively used fluorescence microscopy, which is actually the technique of choice in experiments that require high concentrations of unique DS variants of a human origin. However, our investigations reveal a new, promising experimental model using BT-474 cells and commercially available PM, which enables the effect of DS on the necroptosis induction to be examined using other scientific methods.

## Data Availability

Data will be shared upon request (please contact mkozma@sum.edu.pl).

## Supplementary Materials

The following supporting information can be downloaded at: https://www.mdpi.com/article/10.3390/biom14070829/s1, Figure S1. The DS variants intensified the nuclear translocation of NFκB. Representative images showing the changes in the cellular localization of NFκB in the BT-474 (A) or T-47D (B) cells that were exposed to the DS variants (PM–DS from porcine intestinal mucosa, NF–DS from normal human fascia and DF–DS from fibrosis-affected human fascia) at a concentration of 25 µg/mL for the indicated time periods. NFκB was detected using 1.5 µg/mL of polyclonal antibodies against the human p65 unit (Abcam); the nuclei were stained with Hoechst. Figure S2. The NFκB inhibitor cardamonin (Car) stimulates the DS variant-dependent phosphorylation of MLKL in luminal breast cancer cells. Representative images showing the activation of the necroptotic effector MLKL in the BT-474 (A) and T-47D (B) cells that were first preincubated for 3 h with Car and then exposed to a combination of the inhibitor and individual DS variant for 3.5 h. The activation of MLKL was measured by immunofluorescence. PM–DS from porcine intestinal mucosa, NF–DS from normal human fascia and DF–DS from fibrosis-affected human fascia. Figure S3. Inhibition of the activity of small GTP-ases Rac1 and/or Cdc42 by EHop016 suppresses the DS variant-dependent activation of MLKL in luminal breast cancer cells. Representative images showing the activation of MLKL in the BT-474 (A) and T-47D (B) cells that were first preincubated for 3 h with the indicated concentration of EHop016 and then exposed to a combination of the inhibitor and individual DS variant for 3.5 h. The activation of MLKL was measured by immunofluorescence. PM–DS from porcine intestinal mucosa, NF–DS from normal human fascia and DF–DS from fibrosis-affected human fascia. Figure S4. The universal ROS scavenger N-acetylcysteine amide (NACA) inhibits the DS variant-dependent activation of MLKL in luminal breast cancer cells. Representative images showing the activation of MLKL in the BT-474 (A) and T-47D (B) cells that were first preincubated for 3 h with NACA and then exposed to a combination of the inhibitor and individual DS variant for 3.5 h. The activation of MLKL was measured by immunofluorescence. PM–DS from porcine intestinal mucosa, NF–DS from normal human fascia and DF–DS from fibrosis-affected human fascia. Figure S5. The Rac1/Cdc42 inhibitor EHop016 suppresses the DS variant-mediated induction of the oxidative imbalance in luminal breast cancer cells. Representative images showing the ROS production in the BT-474 (A) and T-47D (B) cells that were first preincubated for 3 h with the indicated concentration of EHop016 and then exposed to a combination of the inhibitor and individual DS variant for 45 min. The ROS production was measured by CellROX Orange. PM–DS from porcine intestinal mucosa, NF–DS from normal human fascia and DF–DS from fibrosis-affected human fascia.

## Abbreviations

CS	chondroitin sulfate
DF	dermatan sulfate from fibrosis-affected palmar fascia
DS	dermatan sulfate
ECM	extracellular matrix
GAG	glycosaminoglycan
MLKL	mixed lineage kinase domain-like pseudokinase
NACA	N-acetylcysteine amide
NF	dermatan sulfate from normal fascia
NFκB	nuclear factor κB
PG	proteoglycan
PM	dermatan sulfate from intestinal mucosa
RIPK	receptor-interacting protein kinase
ROS	reactive oxygen species
TLR	Toll-like receptor
TNF	tumor necrosis factor
