# Supplementary material for: Dermatan Sulfate Affects the Activation of the Necroptotic Effector MLKL in Breast Cancer Cell Lines via the NFκB Pathway and Rac-Mediated Oxidative Stress
Source: Biomolecules. 2024 Jul 10;14(7):829. doi: 10.3390/biom14070829 (PMC11274702; doi:10.3390/biom14070829)
Supplement: Supplementary file 1 [file biomolecules-14-00829-s001.zip › biomolecules-3070688-supplementary.pdf]

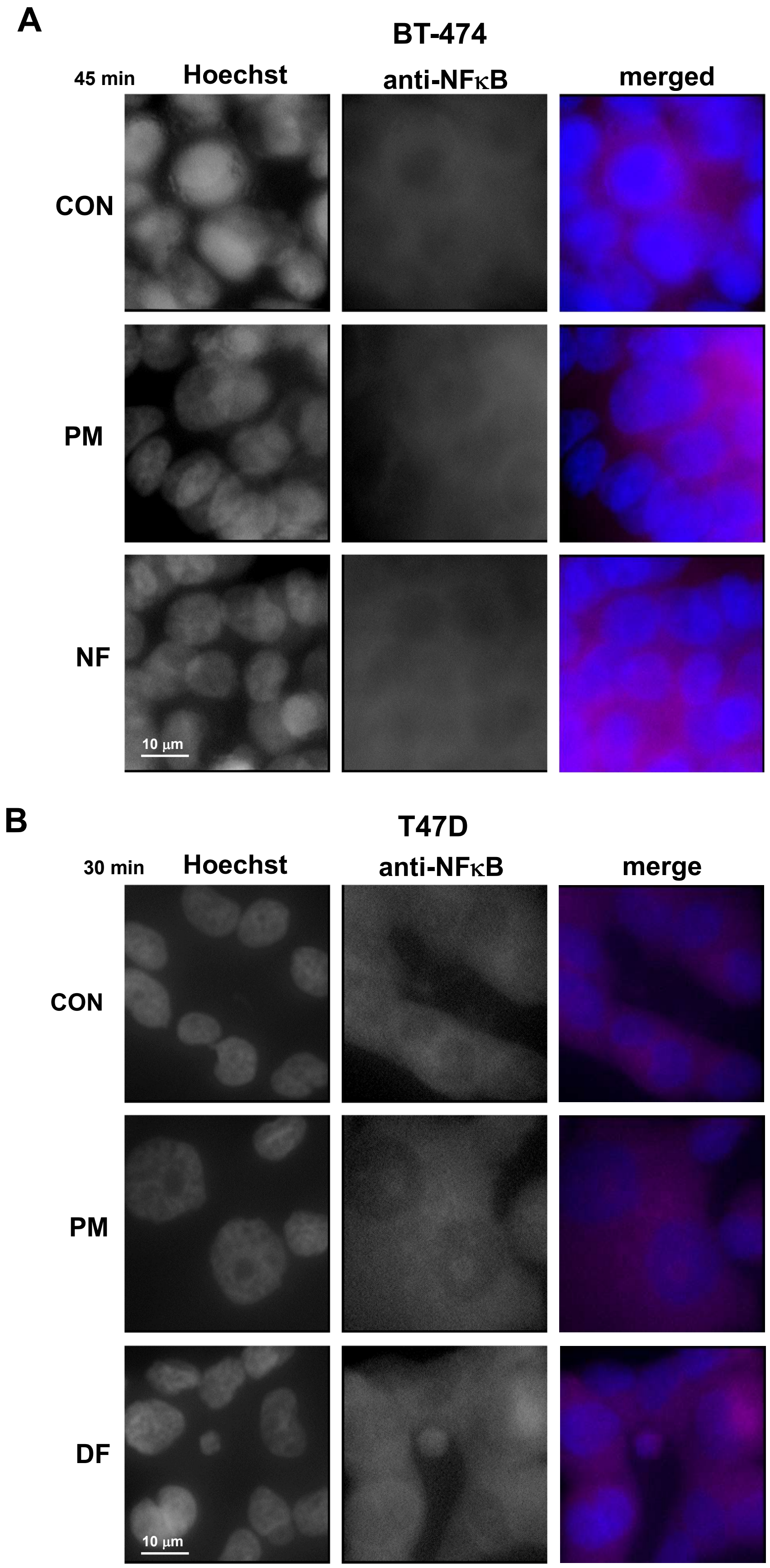
A
BT-474
45 min
Hoechst
anti-NFκB
merged
CON
PM
NF
10 μm
B
T47D
30 min
Hoechst
anti-NFκB
merge
CON
PM
DF
10 μm

Figure S1. The DS variants intensified the nuclear translocation of NFκB. Representative images showing the changes in the cellular localization of NFκB in the BT-474 (A) or T-47D (B) cells that were exposed to the DS variants (PM – DS from porcine intestinal mucosa, NF – DS from normal human fascia, DF – DS from fibrosis-affected human fascia) at a concentration of 25 μg/ ml for the indicated time periods. NFκB was detected using 1.5 μg/ml of polyclonal antibodies against the human p65 unit (Abcam); the nuclei were stained with Hoechst.

A

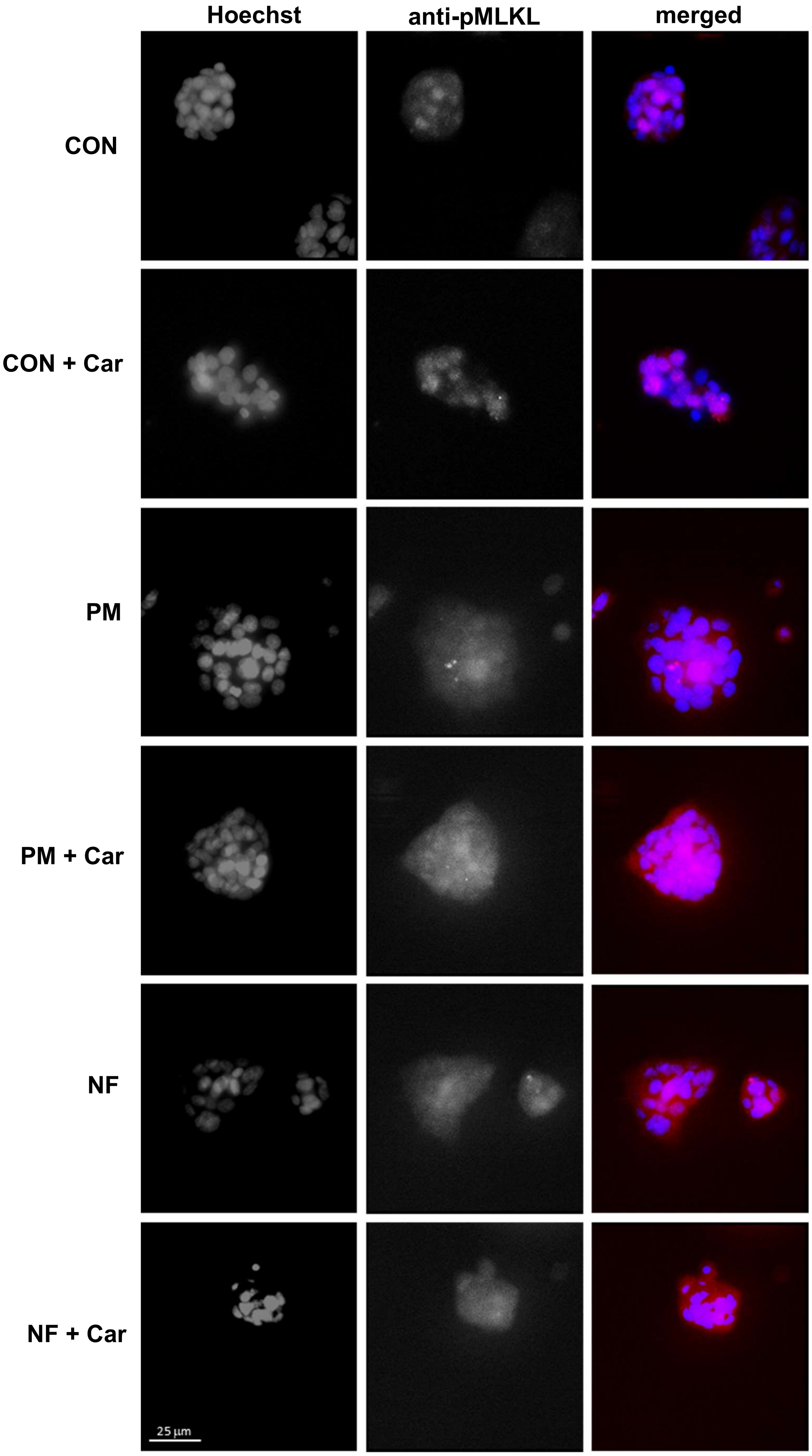

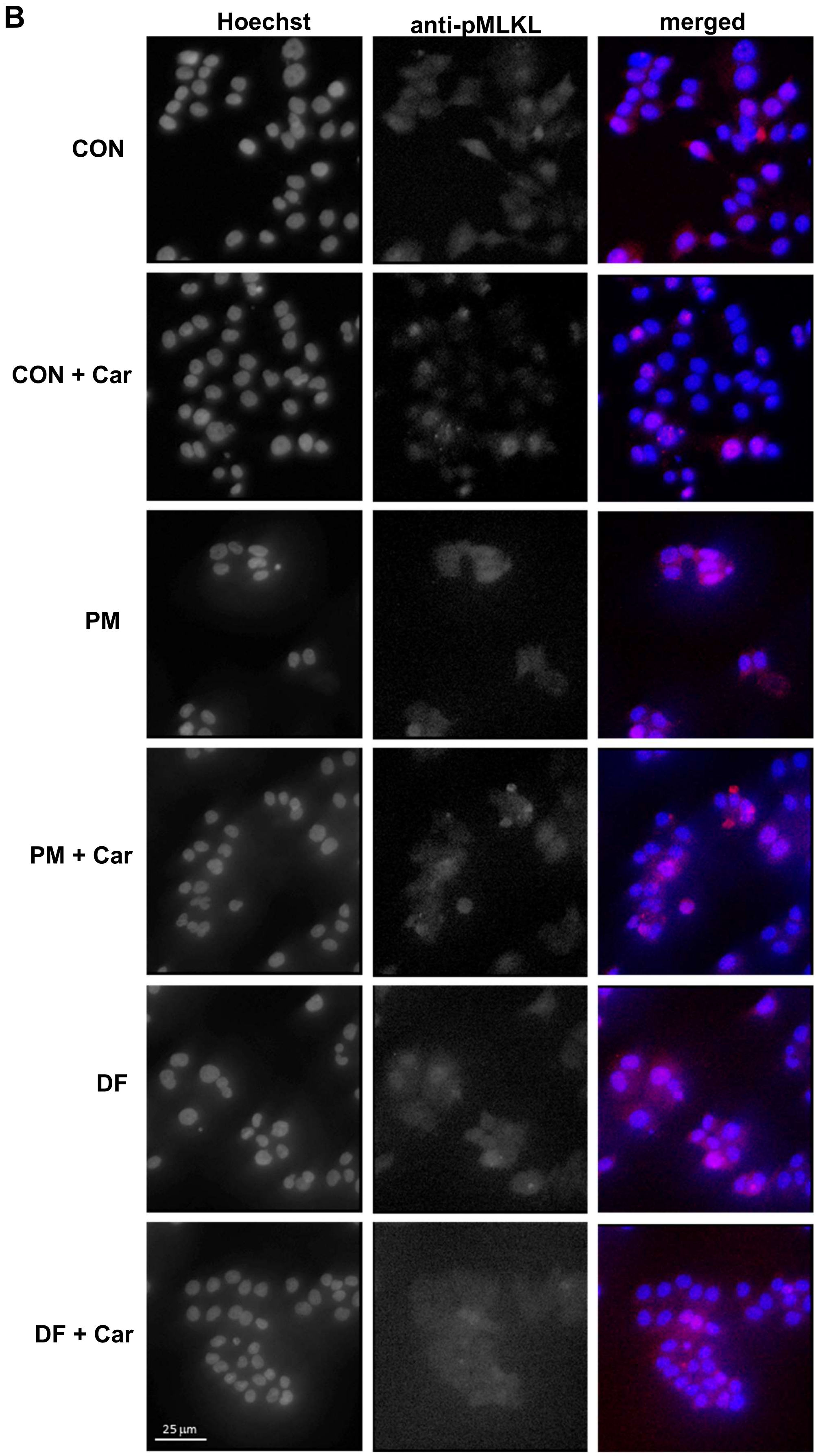

Figure S2. The NFκB inhibitor cardamonin (Car) stimulates the DS variant-dependent phosphorylation of MLKL in luminal breast cancer cells. Representative images showing the activation of necroptotic effector MLKL in the BT-474 (A) and T-47D (B) cells that were first preincubated for 3 h with Car, and then exposed

to a combination of the inhibitor and individual DS variant for 3.5h. The activation of MLKL was measured by immunofluorescence. PM – DS from porcine intestinal mucosa, NF – DS from normal human fascia, DF – DS from fibrosis-affected human fascia.

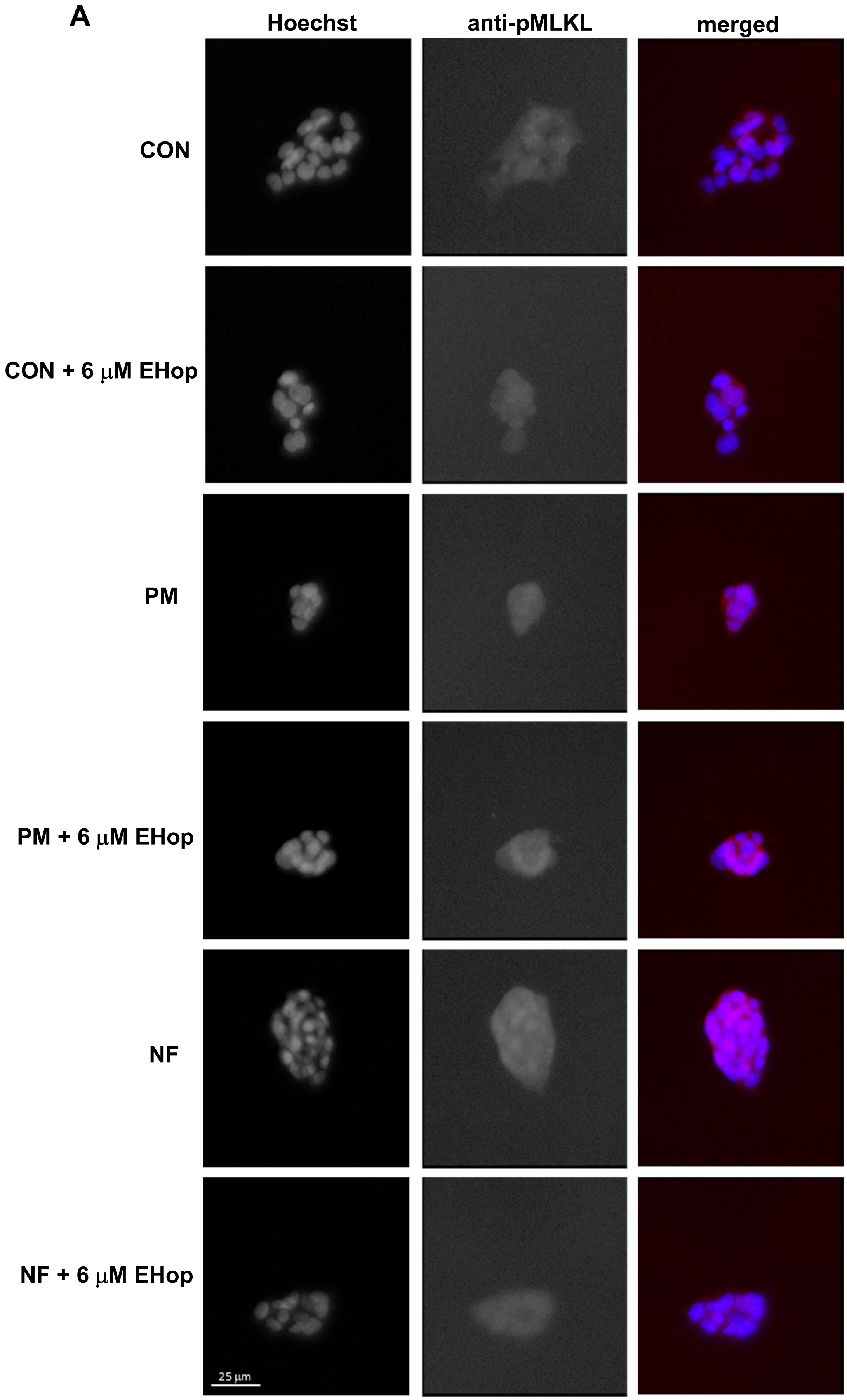

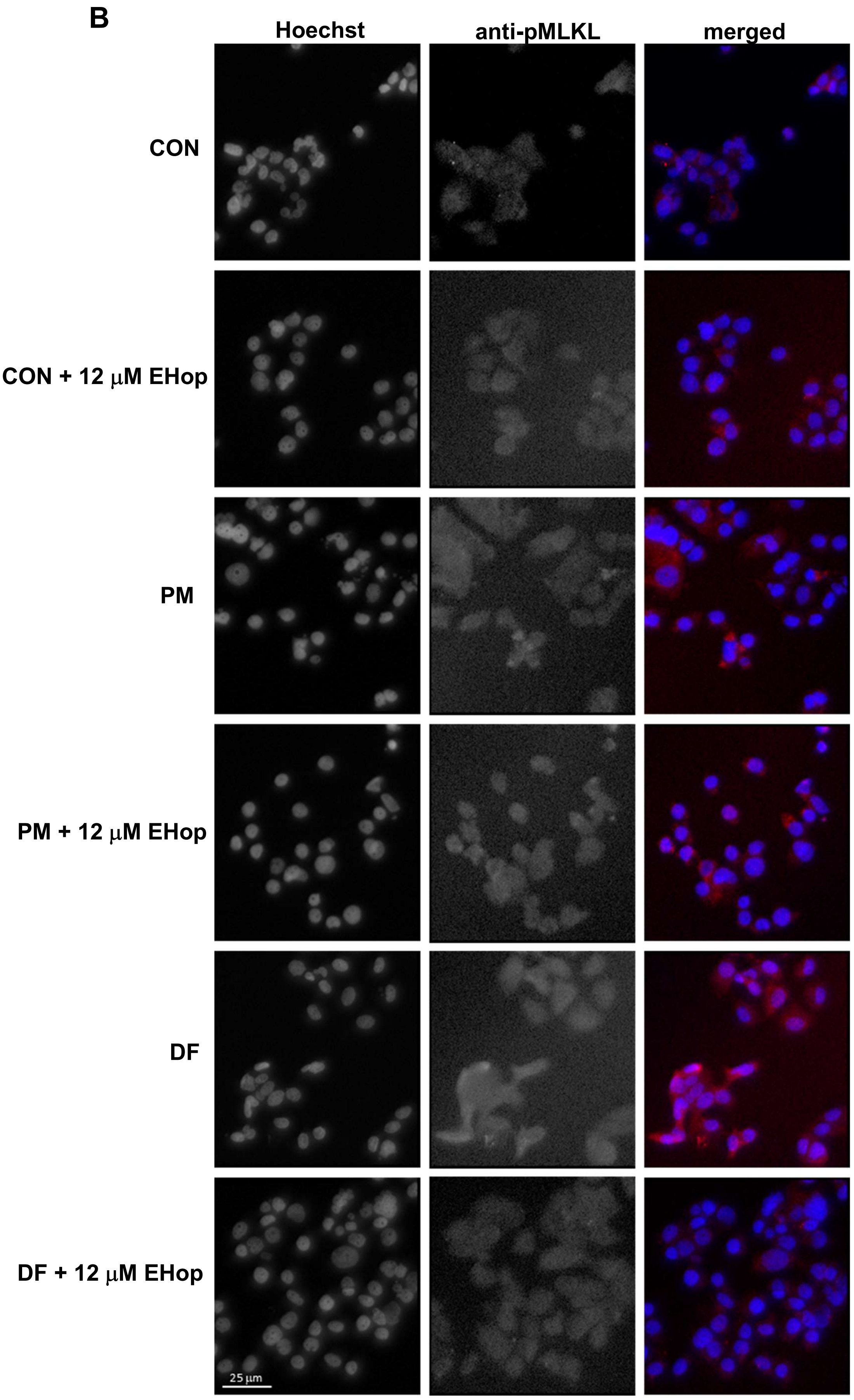

Figure S3. Inhibition of the activity of small GTP-ases Rac1 and/or Cdc42 by EHop016 suppresses the DS variant-dependent activation of MLKL in luminal breast cancer cells. Representative images showing the

activation of MLKL in the BT-474 (A) and T-47D (B) cells that were first preincubated for 3 h with the indicated concentration of EHop016, and then exposed to a combination of the inhibitor and individual DS variant for 3.5h. The activation of MLKL was measured by immunofluorescence. PM – DS from porcine intestinal mucosa, NF – DS from normal human fascia, DF – DS from fibrosis-affected human fascia.

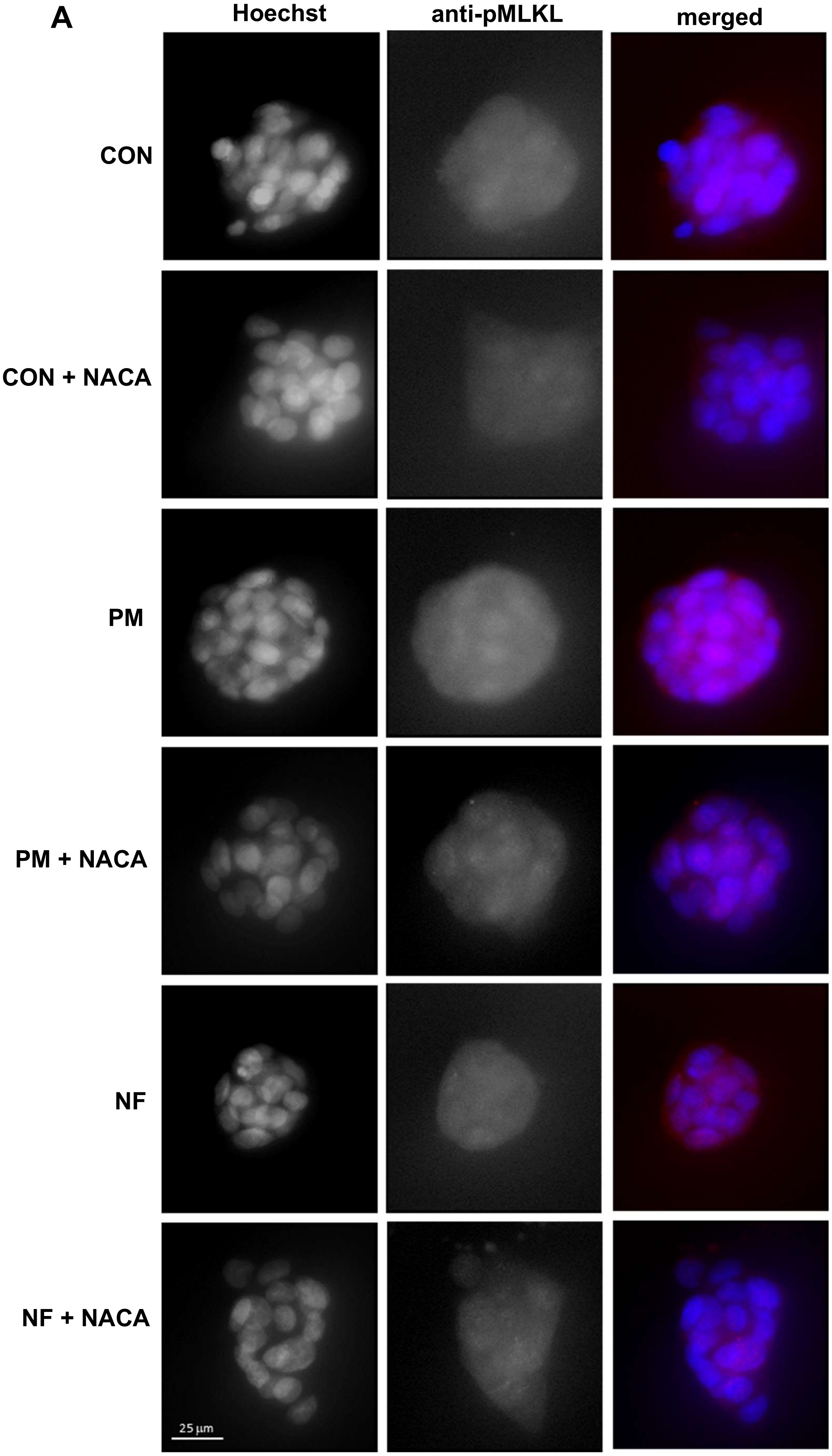

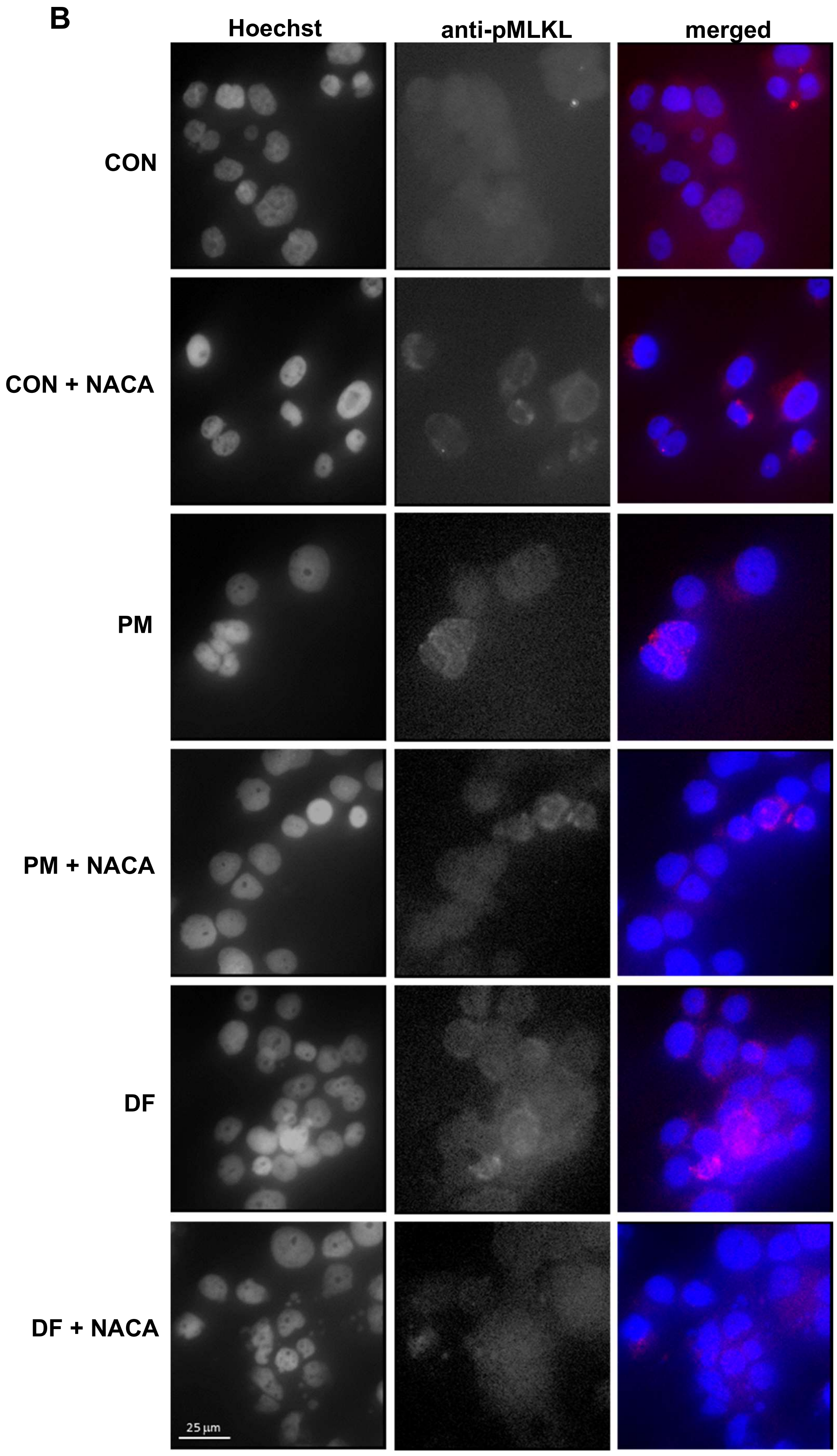

Figure S4. The universal ROS scavenger N-acetylcysteine amide (NACA) inhibits the DS variant-dependent activation of MLKL in luminal breast cancer cells. Representative images showing the activation of MLKL

in the BT-474 (A) and T-47D (B) cells that were first preincubated for 3 h with NACA, and then exposed to a combination of the inhibitor and individual DS variant for 3.5h. The activation of MLKL was measured by immunofluorescence.PM – DS from porcine intestinal mucosa, NF – DS from normal human fascia, DF – DS from fibrosis-affected human fascia.

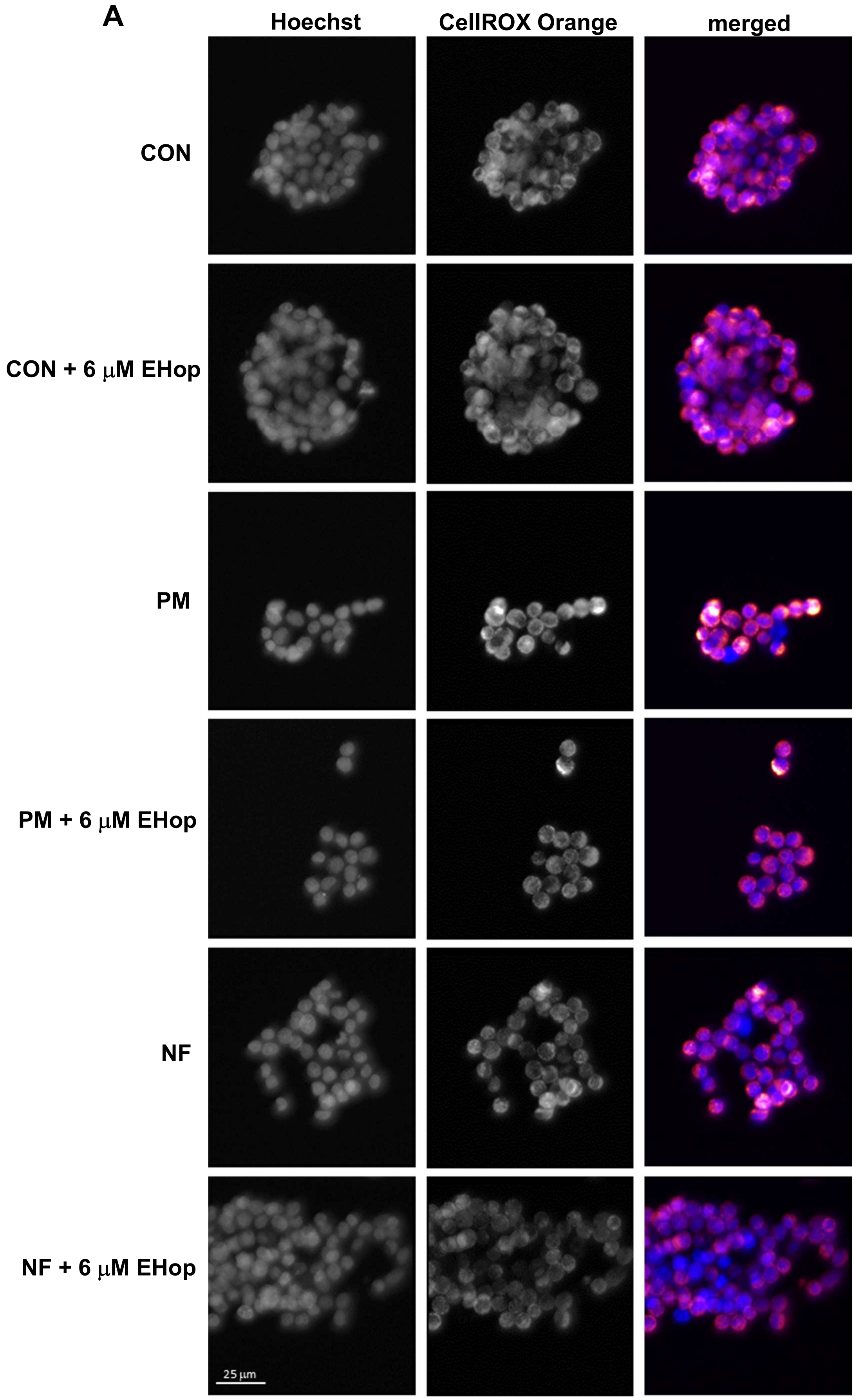

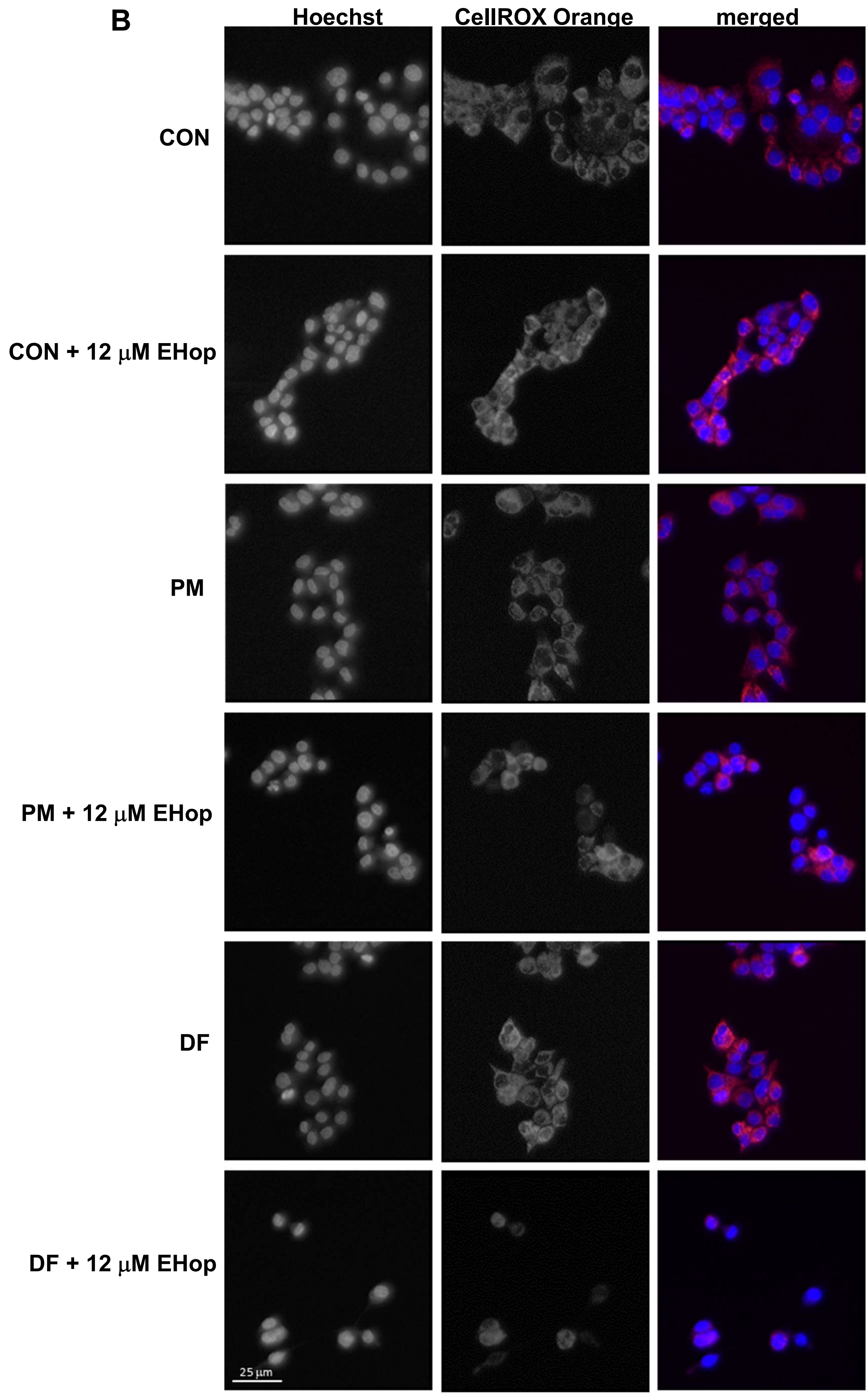

Figure S5. The Rac1/Cdc42 inhibitor EHop016 suppresses the DS variant-mediated induction of oxidative imbalance in luminal breast cancer cells. Representative images showing the ROS production in the BT-474 (A) and T-47D (B) cells that were first preincubated for 3 h with the indicated concentration of EHop016, and

then exposed to a combination of the inhibitor and individual DS variant for 45 min. The ROS production was measured by CellROX Orange. PM – DS from porcine intestinal mucosa, NF – DS from normal human fascia, DF – DS from fibrosis-affected human fascia.
