# Supplementary figures and images for: Dermatan Sulfate Affects the Activation of the Necroptotic Effector MLKL in Breast Cancer Cell Lines via the NFκB Pathway and Rac-Mediated Oxidative Stress
Source: Biomolecules. 2024 Jul 10;14(7):829. doi: 10.3390/biom14070829 (PMC11274702; doi:10.3390/biom14070829)

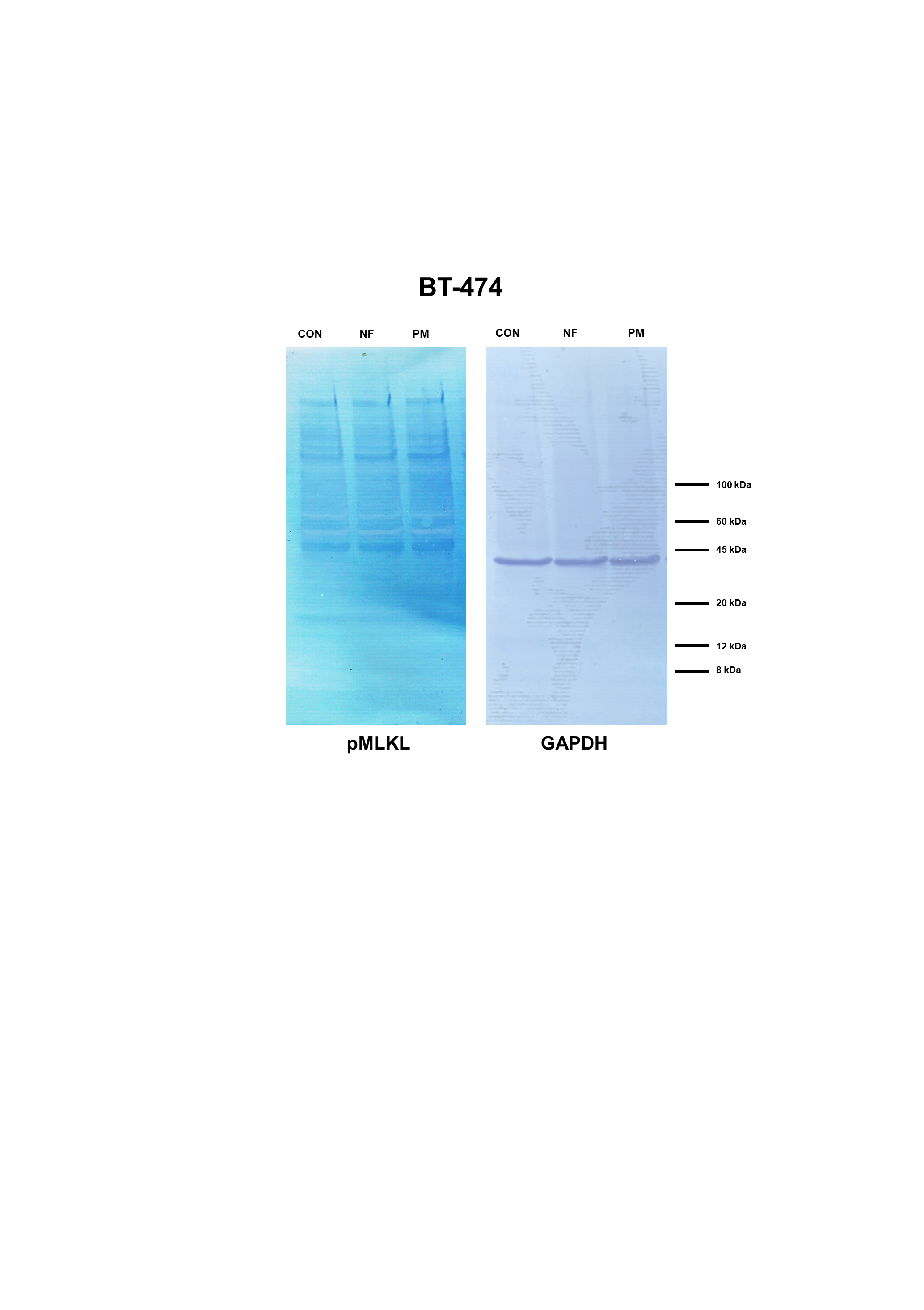

Supplement: Supplementary file 1 [file biomolecules-14-00829-s001.zip › biomolecules-3070688-original images/Fig1B.TIF]

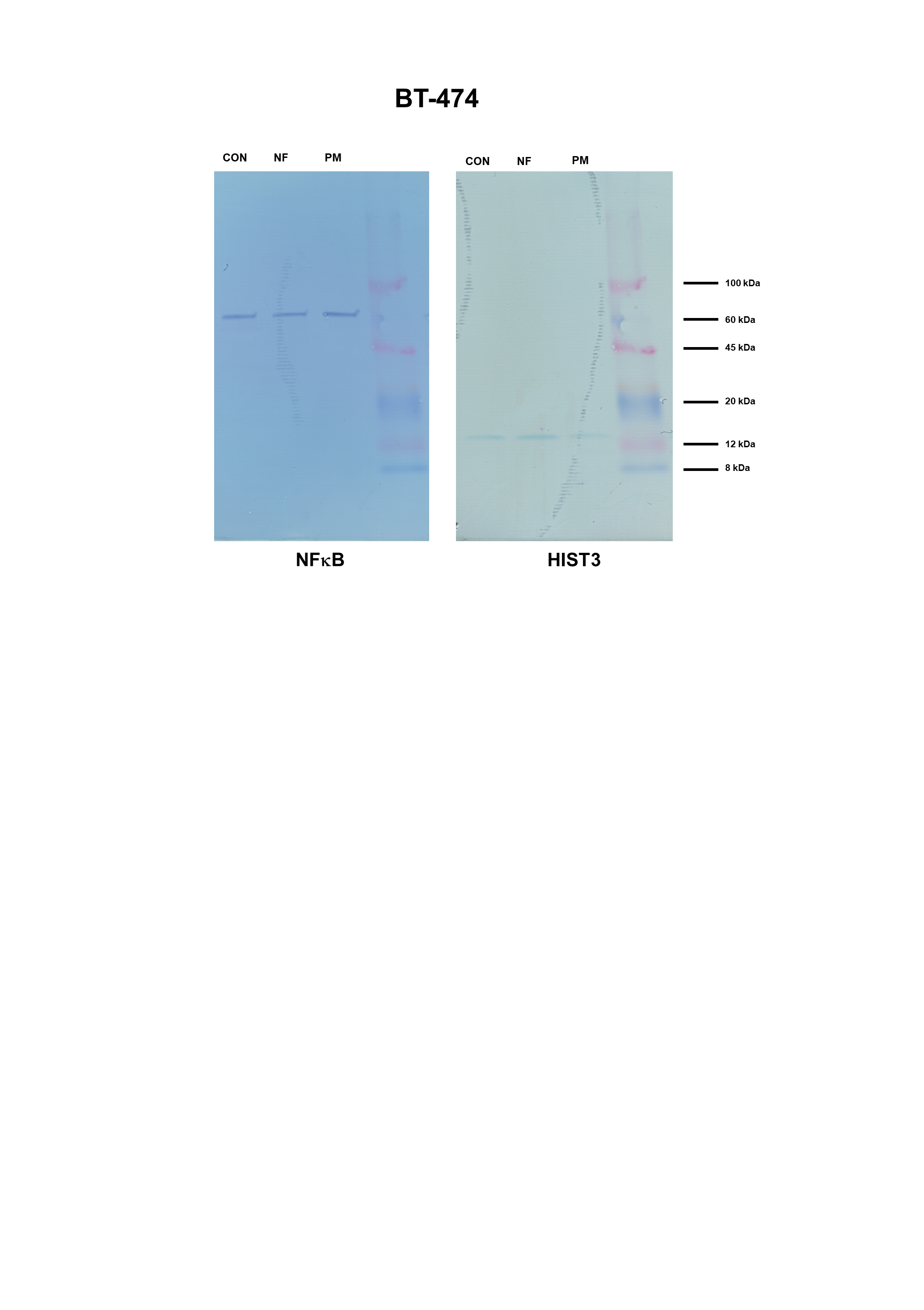

Supplement: Supplementary file 1 [file biomolecules-14-00829-s001.zip › biomolecules-3070688-original images/Fig2C.TIF]

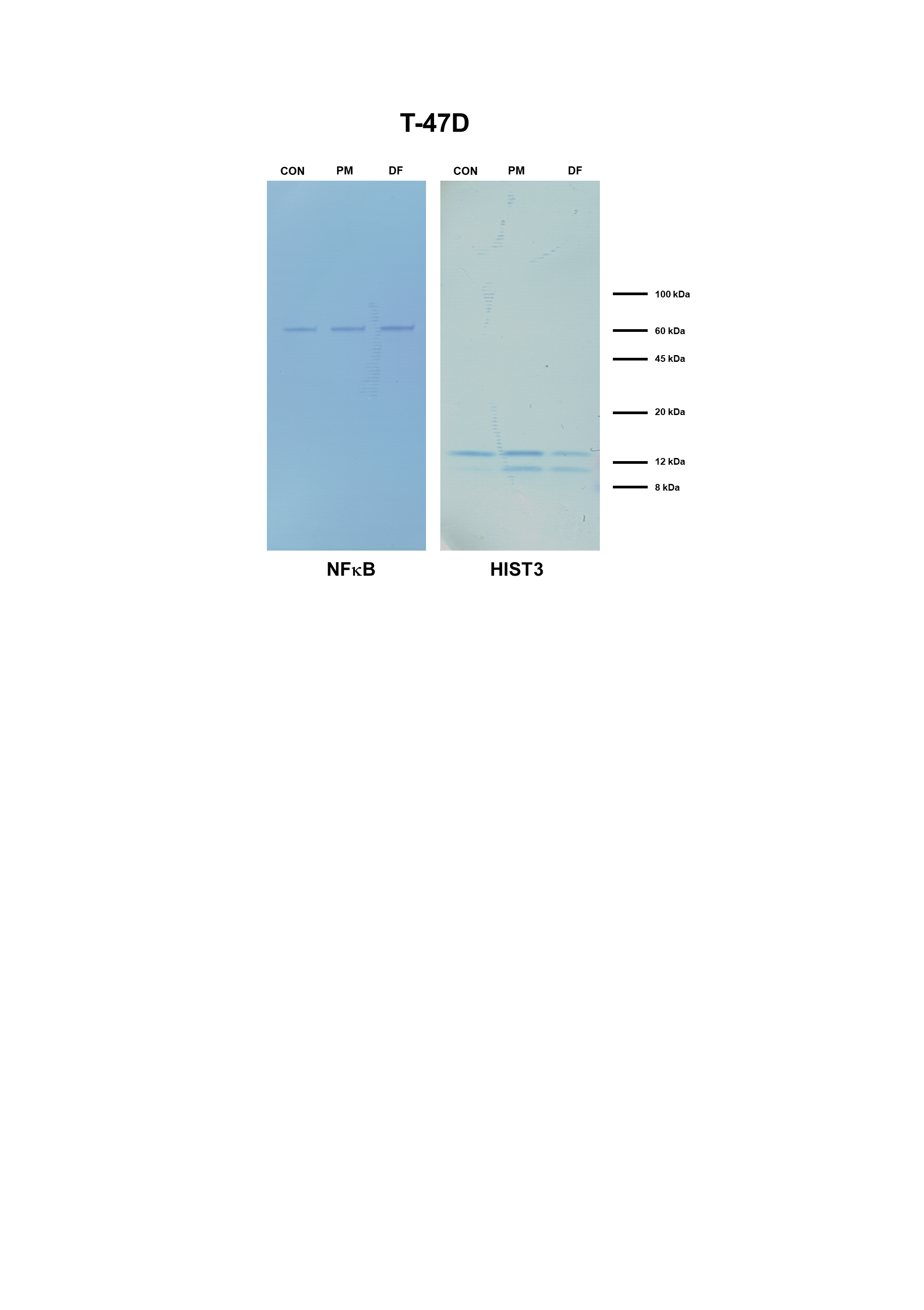

Supplement: Supplementary file 1 [file biomolecules-14-00829-s001.zip › biomolecules-3070688-original images/Fig2E.TIF]
